# Variation in extracellular matrix genes is associated with weight regain after weight loss in a sex-specific manner

**DOI:** 10.1007/s12263-015-0506-y

**Published:** 2015-11-19

**Authors:** Nadia J. T. Roumans, Roel G. Vink, Marij Gielen, Maurice P. Zeegers, Claus Holst, Ping Wang, Arne Astrup, Wim H. Saris, Armand Valsesia, Jörg Hager, Marleen A. van Baak, Edwin C. M. Mariman

**Affiliations:** Department of Human Biology, NUTRIM School of Nutrition and Translational Research in Metabolism, Maastricht University, Universiteitssingel 40, 6200 MD Maastricht, The Netherlands; Department of Complex Genetics, NUTRIM School of Nutrition and Translational Research in Metabolism, Maastricht University, Universiteitssingel 40, 6200 MD Maastricht, The Netherlands; Centre for Health and Society, Institute of Preventive Medicine, Copenhagen University Hospital, Hovedvejen 5, Frederiksberg, 2000 Copenhagen, Denmark; Laboratory of Biochemical Genetics, Department of Clinical Genetics, Maastricht University Medical Centre, University Hospital Maastricht, P. Debyelaan 25, 6229 HX Maastricht, The Netherlands; Department of Nutrition, Exercise and Sports, Faculty of Science, University of Copenhagen, Nørre Allé 51, 2200 Copenhagen, Copenhagen, Denmark; Nestlé Institute of Health Sciences, EPFL Innovation Park, 1015 Lausanne, Switzerland

**Keywords:** Weight regain, Extracellular matrix, SNPs, Adipocytes

## Abstract

The extracellular matrix (ECM) of adipocytes is important for body weight regulation. Here, we investigated whether genetic variation in ECM-related genes is associated with weight regain among participants of the European DiOGenes study. Overweight and obese subjects (*n* = 469, 310 females, 159 males) were on an 8-week low-calorie diet with a 6-month follow-up. Body weight was measured before and after the diet, and after follow-up. Weight maintenance scores (WMS, regained weight as percentage of lost weight) were calculated based on the weight data. Genotype data were retrieved for 2903 SNPs corresponding to 124 ECM-related genes. Regression analyses provided us with six significant SNPs associated with the WMS in males: 3 SNPs in the *POSTN* gene and a SNP in the *LAMB1*, *COL23A1,* and *FBLN5* genes. For females, 1 SNP was found in the *FN1* gene. The risk of weight regain was increased by: the C/C genotype for *POSTN* in a co-dominant model (OR 8.25, 95 % CI 2.85–23.88) and the T/C–C/C genotype in a dominant model (OR 4.88, 95 % CI 2.35–10.16); the A/A genotype for *LAMB1* both in a co-dominant model (OR 18.43, 95 % CI 2.35–144.63) and in a recessive model (OR 16.36, 95 % CI 2.14–124.9); the G/A genotype for *COL23A1* in a co-dominant model (OR 3.94, 95 % CI 1.28–12.10), or the A-allele in a dominant model (OR 2.86, 95 % CI 1.10–7.49); the A/A genotype for *FBLN5* in a co-dominant model (OR 13.00, 95 % CI 1.61–104.81); and the A/A genotype for *FN1* in a recessive model (OR 2.81, 95 % CI 1.40–5.63). Concluding, variants of ECM genes are associated with weight regain after weight loss in a sex-specific manner.

**Electronic supplementary material:**

The online version of this article (doi:10.1007/s12263-015-0506-y) contains supplementary material, which is available to authorized users.

## Introduction

Overweight and obesity have become a worldwide public health problem, associated with increased risk of many health complications such as diabetes and cardiovascular disease (Smyth and Heron 2006). Such risk can be reduced by losing weight (Bessesen 2008). However, remaining at a lower body weight after weight loss provides a challenge for many people (Wing and Phelan 2005). The white adipose tissue plays an important role in the body weight change (Balistreri et al. 2010; Bluher 2013). Weight loss reduces the amount of visceral adiposity and fat mass (Stallone et al. 1991), indicating that adipose tissue is one of the important determinants in this process. It has been proposed that the adipose tissue is also involved in the risk of weight regain after weight loss. White adipocytes are characterised by the presence of a single fat droplet, which almost fills the entire cell, and are surrounded by a thick extracellular matrix (ECM) (Mariman and Wang 2010). The ECM, is known for providing structural support, but also for fulfilling vital roles in cell differentiation, such as the determination, proliferation, polarity, survival, and migration of cells (Hynes 2009; Rozario and DeSimone 2010). Prior studies have noted the importance of the ECM in relation to weight regulation. It was shown that ECM-regulated processes are disturbed in obese mice and humans, leading to the accumulation of immune cells in the adipose tissue, impaired metabolic function, and reduced capacity for fat mass expansion (Divoux et al. 2010; Strissel et al. 2007). After long-term weight reduction, a downregulation of ECM-regulating genes and changes in expression levels of ECM components can be observed in adipose tissue (Divoux et al. 2010; Henegar et al. 2008; Kolehmainen et al. 2008; Kos et al. 2009). In addition, Tam et al. reported that 10 % body weight gain causes an upregulation of ECM-remodelling genes in the adipose tissue of male subjects (Tam et al. 2014). When people decrease their energy intake and enter a negative energy balance, mature adipocytes decrease their fat content and become smaller (Andersson et al. 2014). The ECM is supposed to adjust to changes in cell volume. It has been proposed that this may lead to an improper fit between the cell and the surrounding ECM, thereby inducing tension and cellular stress (Mariman 2011). This cellular stress in adipocytes may be reduced by restoring fat and increasing cell volume, which would mean regain of weight for the host (Mariman 2011, 2012). If the ECM is able to adjust properly to the volume changes, less cellular stress is generated to result in lower risk of weight regain. In line with this view, the subcutaneous adipose tissue ECM gene expression after low-calorie diet has been reported with differences in weight regainers compared to weight maintainers (Mutch et al. 2011). We hypothesise that variation in genes coding for components of the adipocyte ECM are candidates for determining the risk of weight regain or the successfulness of weight maintenance after weight loss. In the present study, we examined whether genetic variation in ECM-related genes is associated with weight regain among the participants of the European DiOGenes study. We analysed the present data separately for males and females because gender specificity has been shown for ECM remodelling in rodents (Lu et al. 2012; Salimena et al. 2004) and humans (Bowers et al. 2010; Komosinska-Vassev et al. 2011), although it has not been specifically examined in adipose tissue.

## Materials and methods

### Participants and study design

Participants took part in a pan-European, multicentre, randomised controlled dietary intervention programme called DiOGenes (http://www.diogenes-eu.org, ClinicalTrials.gov registration no.: NCT00390637). The whole study design has been described in detail previously (Larsen et al. 2010a, b; Wang et al. 2012). This study was conducted in eight European countries: the Netherlands, Denmark, the UK, Greece, Bulgaria, Germany, Spain, and the Czech Republic. For 8 weeks, healthy overweight or obese participants followed a low-calorie diet that provided about 3.3–4.2 MJ/day which is between 800 and 1000 kcal/day. After the diet, participants were randomly assigned to 1 of 4 ad libitum consumed low-fat weight maintenance diets. These diets differed in glycemic index and protein content (Larsen et al. 2010b). Body weight and other physical and biochemical parameters were measured after overnight fasting on a calibrated scale before weight loss on clinical investigation day 1 (CID1, *t* = 0), after LCD on CID2 (*t* = 8*w*), and after weight maintenance on CID3 (*t* = 8*w* + 6*m*). For the current analysis, only participants who provided weight measurements at all 3 investigation days and who were successfully genotyped were used. In total, 469 participants met these criteria. Weight maintenance scores were calculated for all 469 participants as previously described by Wang et al. (2012):

$$\begin{aligned} {\text{WMS}} & = \left( {{\text{weight}}\,{\text{at}}\,{\text{CID}}3 - {\text{weight}}\,{\text{at}}\,{\text{CID2}}} \right) \\ & \quad \div \left( {{\text{weight}}\,{\text{at}}\,{\text{CID}}1 - {\text{weight}}\,{\text{at}}\,{\text{CID}}2} \right) \\ \end{aligned}$$

A score equal or lower than zero indicated that the participant maintained or continued to lose weight (WM) during the follow-up period, while a score higher than zero indicated that the participant regained weight (WR) during the 6-month follow-up.

### DNA extraction and genotyping

Buffy coats of EDTA-blood were used to extract DNA for genotyping. Genotyping was done using the Illumina Bead Station System (IlluminaInc) by IntegraGen using the Illumina 660quad chip, which analyses 660.000 SNPs and CNVs per sample. Genotype QC was carried out for all SNPs, and SNPs were excluded from the analysis if they had a call rate <98 %, MAF < 0.01 or were not in HWE. Centre d’Etude du Polymorphisme Humain control samples were added on each plate: one was different on each plate, and one was identical among the 15 genotyped plates. The reproducibility was 100 %, and the concordance rate was 99.6 %. For the purpose of the present study, genotypes for all individuals were extracted from the genotyping matrix for the candidate SNPs only.

Based on the proteins detected in the adipocyte ECM (Mariman and Wang 2010), a list of 124 candidate genes related to the ECM was created (Supplement Table S1). Genotype data from the DiOGenes cohort were retrieved for 2903 SNPs (Supplement Table S2) in and near the 124 genes.

### Data analysis

Dependant *t* test was applied to check for differences between time points within a group. A Chi-square test was used to check whether the genotype frequencies of the SNPs were in Hardy–Weinberg equilibrium.

Univariate linear regression analyses were carried out with each SNP allele as a predictor and weight maintenance scores as outcome. The analyses were done for males and females separately because of gender specificity in ECM remodelling found in other studies. Regression analyses were done with the use of Stata 12.0 (StataCorp LP). The *P* values were corrected for false discovery rate (FDR) in multiple testing with the Benjamini–Hochberg method with the ‘stats’ package in R (version 3.1.1; http://www.r-project.org/) (Benjamini and Hochberg 1995). Corrected *P* values <0.05 were considered to be statistically significant. Next, SNPs with a minor allele frequency <5 % were excluded to distinguish common polymorphism from rare variants. Linkage disequilibrium *r*^2^ values >0.2 was used to determine whether periostin SNPs were in linkage disequilibrium. Linkage disequilibrium structure was evaluated by using SNPStats (Sole et al. 2006).

Backward elimination in multivariate linear regression was used to check whether combinations of SNPs might enhance the outcome. For this, the four significant SNPs observed in the male population were used.

Genotype analyses: logistic regression analysis was used to find the best genetic model of inheritance that describes the effect of the genotypes of the significant SNPs. A model is considered best fitting if it has the lowest Akaike information criterion (AIC) score and if this value is at least 2 lower than the other models. If multiple models have similar low AIC values than these models are fitting equally well, logistic regression, odds ratios (OR), and 95 % confidence intervals (95 % CI) were calculated to determine the risk of a specific genotype on weight regain. Genotype analyses were all carried out using Stata 12.0 (StataCorp LP).

## Results

### Participant characteristics

Participant characteristics of weight regainers and maintainers, separated for males and females, can be seen in Table 1. Comparisons between baseline and after 8 weeks show that weight, BMI, WC, and fat mass (FM) significantly decreased after the 8-week low-calorie diet (LCD) for all groups. After the 6-month weight maintenance period, all parameters were significantly decreased for weight maintainers (WM), while a significant increase is observed for weight regainers (WR) when comparing to measurements after the LCD diet. Weight maintenance scores (WMS) were significantly different between WM and WR for males (*P* < 0.001) and females (*P* < 0.001).

**Table 1:** Changes in subject characteristics at the end of 8-week low-calorie diet compared to baseline, and at the end of 6-month ad libitum diet compared to the end of 8-week low-calorie diet

	Baseline				After 8-week LCD				After 6-month ad libitum diet			
	Male		Female		Male		Female		Male		Female	
	WM	WR	WM	WR	WM	WR	WM	WR	WM	WR	WM	WR
*n*	59	100	135	175	59	100	135	175	59	100	135	175
Age (year)	43 ± 6	43 ± 6	41 ± 7	42 ± 6								
Weight (kg)	111.3 ± 18.5	107.9 ± 17.1	97.3 ± 18.3	92.1 ± 13.1	97.7 ± 17.3*	95.3 ± 15.0*	86.3 ± 16.6*	82.6 ± 12*	93.5 ± 17.5^#^	99.8 ± 15.7^#^	81.6 ± 15.1^#^	86.2 ± 12.6^#^
BMI (kg/m^2^)	35.4 ± 4.9	33.4 ± 4.6	35.1 ± 5.4	33.5 ± 4.4	31.1 ± 4.6*	29.6 ± 4.1*	31.1 ± 4.8*	30.1 ± 4.0*	29.8 ± 4.7^#^	30.9 ± 4.3^#^	29.5 ± 4.5^#^	31.4 ± 4.2^#^
WC (cm)	117.4 ± 12.9	111.9 ± 11.8	104.9 ± 13.6	102.3 ± 10.4	105.4 ± 12.7*	100.5 ± 11.5*	95.2 ± 12.3*	93.6 ± 10.1*	101 ± 12.4^#^	104.5 ± 12.0^#^	91.8 ± 12.2^#^	96.4 ± 10.1^#^
Fat mass (kg)	39.6 ± 13.4	34.1 ± 10.7	44 ± 12	40.8 ± 9.3	30.4 ± 10.6*	26.7 ± 10.1*	35.3 ± 10.9*	33.8 ± 9.3*	25.3 ± 10.6^#^	28.4 ± 10.3^#^	30.5 ± 9.4^#^	35.6 ± 9.2^#^
WMS									−0.33 ± 0.34	0.38 ± 0.25	−0.42 ± 0.39	0.40 ± 0.31

Values are mean ± SD. Subjects are divided into four groups: male weight maintainers (WM, *n* = 59), weight regainers (WR, *n* = 100) and female WM (*n* = 135) and WR (*n* = 175). Weight maintenance score (WMS) is calculated at the end of the 6-month ad libitum diet: (weight after 6-month ad libitum diet − weight after LCD)/(weight at baseline − weight after LCD)

*WC* waist circumference, *LCD* low-calorie diet, *WMS* weight maintenance score

* *P* < 0.001 change from baseline versus after 8-week LCD with dependant *t* test per group

^#^ *P* < 0.001 change from after 8-week LCD versus after 6-month ad libitum diet with dependent *t* test per group

### Single-nucleotide polymorphism univariate linear regression analyses

The single-nucleotide polymorphisms (SNPs) with a corrected *P* value <0.05 are depicted in Table 2, and the results of all SNPs are shown in Table S1. Further selection on minor allele frequency (MAF) resulted for the male group in only 6 SNPs with an allele frequency higher than 5 % (indicated in bold in Table 2): rs7323378, rs9315503, rs9547947, rs2158836, rs12589592, and rs2672826. Three of the SNPs are in and around the periostin gene (*POSTN*, rs7323378, rs9315503, rs9547947), and the other SNPs are in the laminin-β1 (*LAMB1*, rs2158836), fibulin-5 (*FBLN5*, rs12589592), and collagen, Type XXIII, alpha1 **(***COL23A1*, rs2672826) genes. The three *POSTN* SNPs were all in linkage disequilibrium: rs7323378–rs9547947 (*r*^2^ = 0.760), rs7323378–rs9315503 (*r*^2^ = 0.487), and rs9547947–rs9315503 (*r*^2^ = 0.370). It indicates that these variants are closely linked, and therefore, only the SNP with the lowest *P* value was used for further analysis, which is *POSTN* rs7323378. For female subjects, 1 SNP remained after selecting SNPs with a minor allele frequency higher than 5 %: rs17516906. This SNP is located in the fibronectin 1 (*FN1*) gene.

**Table 2:** Regression analyses carried out with each SNP allele as a predictor and weight maintenance scores as outcome separately for males and females

SNP	Gene	No. of subjects	*P* value	*P*_corr_	Minor allel	MAF %
*Males*						
rs8031741	ACAN	159	9.57E−26	<0.001	G	0.3
rs2271649	ADAM12	158	5.12E−07	<0.001	A	0.9
rs16859850	CCDC80	158	4.08E−17	<0.001	G	0.6
rs2300792	COL12A1	157	1.43E−22	<0.001	C	0.3
rs16918099	COL15A1	159	3.29E−05	0.005	A	0.2
rs16918124	COL15A1	159	6.19E−05	0.008	C	0.5
rs7863250	COL15A1	159	6.19E−05	0.008	C	0.5
**rs2672826**	**COL23A1**	**157**	**4.74E−04**	**0.048**	**A**	**12.1**
rs12477499	COL3A1	159	2.49E−14	<0.001	G	0.3
**rs12589592**	**FBLN5**	**159**	**2.13E−04**	**0.023**	**A**	**33.1**
rs12050562	FBN1	159	8.75E−05	0.010	T	0.8
rs7606877	GPC1	159	1.41E−14	<0.001	A	0.5
rs9492168	LAMA2	158	7.50E−17	<0.001	T	0.3
**rs2158836**	**LAMB1**	**159**	**1.75E−04**	**0.020**	**A**	**37.1**
rs10911215	LAMC1	159	1.11E−09	<0.001	T	1.3
rs2513812	MATN2	159	4.36E−09	<0.001	G	1.3
rs1151578	NID2	158	2.53E−08	<0.001	T	0.2
rs6480654	P4HA1	157	2.67E−12	<0.001	A	0.2
rs1382192	PDIA4	159	4.90E−13	<0.001	A	0.5
rs4727007	PDIA4	159	4.90E−13	<0.001	G	0.5
rs10197695	PDIA6	159	1.26E−14	<0.001	G	0.3
**rs7323378**	**POSTN**	**158**	**1.10E−05**	**0.002**	**C**	**48.0**
**rs9547947**	**POSTN**	**149**	**2.62E−05**	**0.004**	**A**	**38.3**
**rs9315503**	**POSTN**	**159**	**2.20E−04**	**0.023**	**G**	**33.9**
rs7679471	TLL1	159	3.36E−07	<0.001	C	0.2
*Females*						
rs8031741	ACAN	309	5.14E−16	0.000	G	0.3
rs4871046	COL14A1	307	5.49E−09	0.000	C	0.5
rs16918099	COL15A1	310	5.75E−44	0.000	A	0.2
rs12477499	COL3A1	310	2.19E−43	0.000	G	0.3
**rs17516906**	**FN1**	**303**	**1.28E−04**	**0.040**	**G**	**7.4**
rs1151578	NID2	310	5.19E−06	0.002	T	0.2
rs11925421	PLOD2	310	7.20E−10	0.000	G	0.6
rs7078493	TLL2	310	5.19E−06	0.000	T	0.2
rs310517	VCAN	310	1.79E−25	0.000	T	0.5

*P* values are derived from univariate linear regression analyses, and *P*_corr_ are *P* values corrected for false discovery rate. *P*_corr_ values <0.05 are considered significant, and SNPs with a MAF < 5 % are excluded. SNPs in bold have *P*_corr_ values <0.05 and MAF > 5 %

*MAF* minor allel frequency

### SNP multivariate linear regression analyses

Backward elimination in multivariate linear regression with the significant SNPs observed in the male population resulted in significance for three SNPs: rs2672826 (*β* = −17.52, *P* = 0.020), rs2158836 (*β* = −11.50, *P* = 0.039), and rs7323378 (*β* = −13.67, *P* = 0.009). This indicates that *COL23A1*, *POSTN,* and *LAMB1* together have an additive effect on weight regulation.

### Genotype analyses

**Fig. 1:**
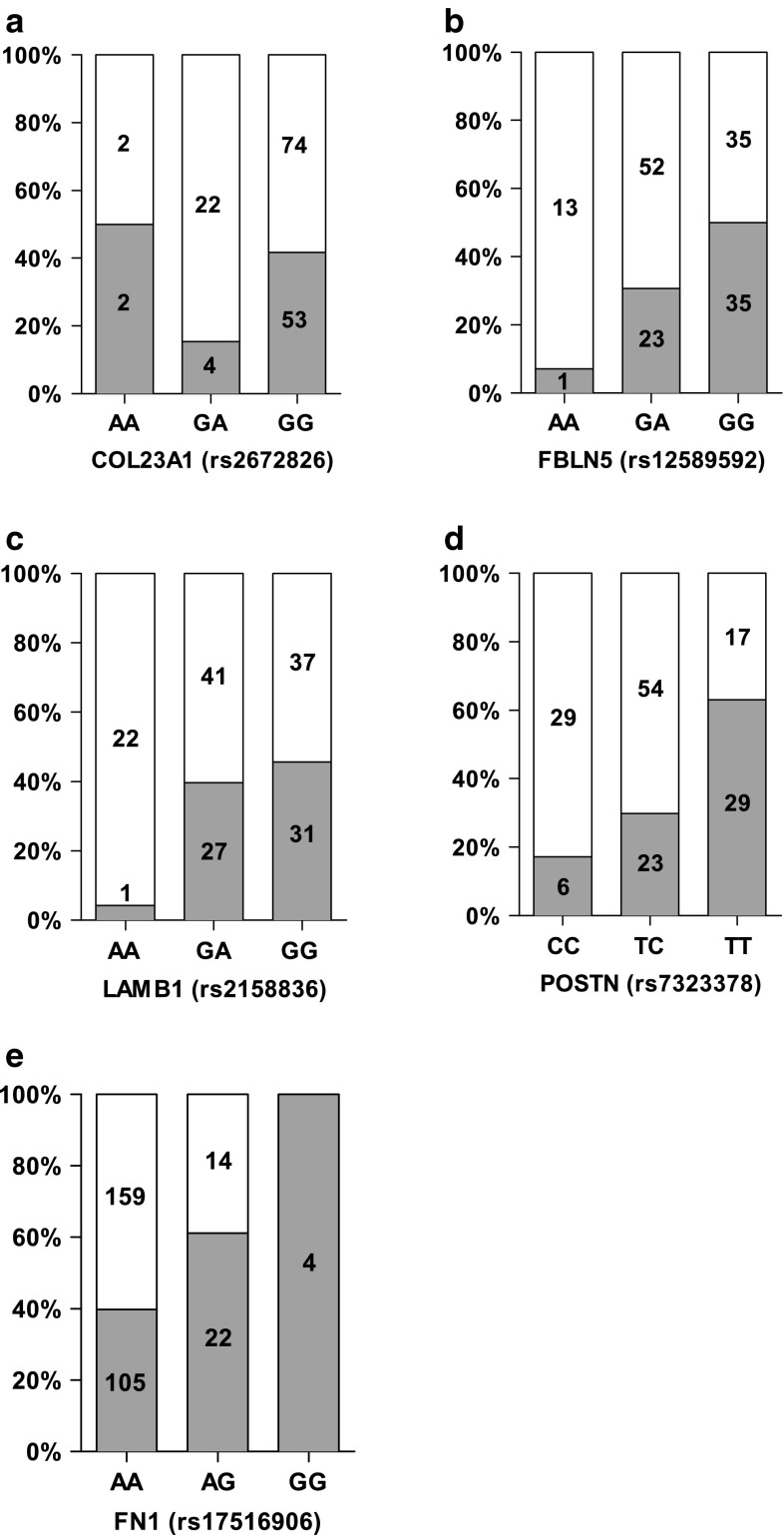
Percentage weight regain or maintenance phenotype of each genotype for the significant SNPs. Each *bar* represents the total amount of subjects having a specific genotype for a significant SNP: **a** COL23A1 (rs2672826), **b** FBLN5 (rs12589592), **c** LAMB1 (rs2158836), **d** POSTN (rs7323378), and **e** FN1 (rs17516906). **a–d** represent men, and **e** represents women. The *grey bars* indicate the percentage of weight maintainers (WM) with the genotype, and the *white bars* are for weight regainers (WR). The number within each *bar* is the count of participants having the specific genotype

Genotype analyses were done to get an idea of the effect of specific genotypes on weight change. Figure 1 shows the comparison between a number of genotypes of weight maintainers (grey bars) and weight regainers (white bars) for the significant SNPs. Table 3 shows the best model of fit and the associations between genotypes and the risk of weight regain. For *COL23A1* rs2672826, the best-fitting models are the co-dominant and dominant models. In the co-dominant model, it is seen that a G/A genotype increases the risk of weight regain by 3.9 times compared to a G/G genotype (OR 3.94, 95 % CI 1.28–12.10). The dominant model indicates that the G/A–A/A genotype increases the risk of weight regain by 2.9-fold compared to the G/G genotype (OR 2.86, 95 % CI 1.10–7.49). The best-fitting model for *FBLN5* rs12589592 is the co-dominant model with a 13-fold increased risk of weight regain with an A/A genotype (OR 13.00, 95 % CI 1.61–104.81) and a 2.3-fold increase with a G/A genotype (OR 2.26, 95 % CI 1.15–4.46) compared to a G/G genotype. The best-fitting models for *LAMB1* rs2158836 are the co-dominant and the recessive models. In the co-dominant model, an A/A genotype gives a 18.4 times higher chance for weight regain than a G/G genotype (OR 18.43, 95 % CI 2.35–144.63). In the recessive model, this risk is increased 16.4 times (OR 16.36, 95 % CI 2.14–124.9). The best-fitting models for *POSTN* rs7323378 are the co-dominant as well as the dominant model. The chance for weight regain is increased 8.3 times with the C/C genotype in the co-dominant model (OR 8.25, 95 % CI 2.85–23.88) when compared to a T/T genotype. In the dominant model, a T/C–C/C genotype increases the risk of weight regain 4.9 times (OR 4.88, 95 % CI 2.35–10.16). The co-dominant model and recessive model are best fitting for the *FN1* rs17516906 SNP but a correct comparison in the co-dominant model cannot be made due to the absence of G/G genotypes for participants regaining weight during follow-up. The recessive model shows that an A/A genotype increases the risk of weight regain 2.8 times (OR 2.81, 95 % CI 1.40–5.63) compared to a G/G–A/G genotype.

**Table 3:** Logistic regression analyses used to find the best genetic model of inheritance that describes the effect of the genotypes

SNP	Model	Genotype	OR (95 % CI)	AIC
rs2672826 A>G COL23A1	Co-dominant	G/G G/A A/A	1.00 3.94 (1.28–12.10)* 0.72 (0.10–5.25)	206.4
	Dominant	G/G G/A–A/A	1.00 2.86 (1.10–7.49)*	206.6
	Recessive	G/G–G/A A/A	1.00 0.59 (0.08–4.33)	211.6
rs12589592 G>A FBLN5	Co-dominant	G/G G/A A/A	1.00 2.26 (1.15–4.46)* 13.00 (1.61–104.81)*	202.7
	Dominant	G/G G/A–A/A	1.00 2.71 (1.40–5.25)*	204.8
	Recessive	G/G–G/A A/A	1.00 8.67 (1.10–68.06)*	206.4
rs2158836 A>G LAMB1	Co-dominant	G/G G/A A/A	1.00 1.27 (0.64–2.51) 18.43 (2.35–144.63)*	199.3
	Dominant	G/G G/A–A/A	1.00 1.89 (0.98–3.62)*	210.1
	Recessive	G/G–G/A A/A	1.00 16.36 (2.14–124.9)*	197.8
rs7323378 T>C POSTN	Co-dominant	T/T T/C C/C	1.00 4.01 (1.85–8.67) 8.25 (2.85–23.88)*	192.6
	Dominant	T/T T/C–C/C	1.00 4.88 (2.35–10.16)*	192.7
	Recessive	T/T–T/C C/C	1.00 3.54 (1.37–9.14)*	203.6
rs17516906 A>G FN1	Co-dominant	G/G A/G A/A	1.00 0.00 (NA) 0.00 (NA)	409.0
	Dominant	G/G A/G–A/A	1.00 0.00 (NA)	412.8
	Recessive	G/G–A/G A/A	1.00 2.81 (1.40–5.63)*	410.7

Odds ratio, 95 % confidence interval, Akaike information criterion (AIC), and *P* values were retrieved from logistic regression analyses with the co-dominant, dominant, and recessive models. The best-fitting model has the lowest AIC, and this value is at least 2 lower than the other models. *P* values <0.05 are considered significant (*)

*OR* odds ratio, *CI* confidence interval, *NA* not available

## Discussion

The major finding of this study is that variants of the *POSTN*, *LAMB1,COL23A1,* and *FBLN5* genes for males and the *FN1* gene for females can influence the risk of weight regain. To the best of our knowledge, this is the first study that relates genetic variation in ECM genes with the risk of weight regain after weight loss. As such, our findings are in keeping with the proposed prominent role of the ECM in the adipocyte cellular stress model for weight regain (Mariman 2011).

For most of the identified genes or members of the same gene family, links with human weight regulation have been reported. POSTN is highly expressed in collagen-rich connective tissue such as adipose tissue. It has been related to obesity by Bolton et al. who observed higher POSTN expression in the adipose tissue of obese diabetic sand rats (*Psammomys obesus*) compared to healthy lean animals, suggesting that POSTN may influence fat storage in adipocytes (Bolton et al. 2009). High expression of POSTN was found in visceral as well as subcutaneous adipose tissue depots, and a role in repair or expansion of the adipose tissue was suggested (Bolton et al. 2009). Proper biomechanical function of connective tissue seems to depend on the interaction with POSTN and specific collagens (Norris et al. 2007). In *POSTN*-knockout mice, a decreased collagen cross-linking was observed and the mechanical stabilisation of ECM architecture was disrupted (Norris et al. 2007). Here we show that in humans the C/C genotype of the *POSTN* SNP rs7323378 increases the risk of weight regain after weight loss, which supports a role for the ECM in human weight regulation.

Laminins constitute a family of 12 genes coding for ECM components, which are subdivided in five α-, four β-, and three γ-genes. SNPs in the *LAMA5* gene have been associated with adiposity parameters in European and African Americans (De Luca et al. 2008). In addition, a rare variant with a moderate-to-high predicted biological effect was detected in *LAMC1* or *LAMC3* in 5 of 30 extremely obese subjects (Mariman et al. 2014). In the present study, we observed that the minor A/A genotype for the *LAMB1* SNP increases the chance of weight regain after weight loss. Laminins actually form trimers from an α, β, and γ protein. Of all possible combinations, only fifteen different trimers have been observed in vivo. LAMB1 is a component of six of those fifteen, where it occurs in combination with LAMC1 or LAMC3.

Collagen XXIII belongs to the nonfibrillar transmembranous subfamily of collagens, and as such it can be involved in cell–matrix contact, but concrete information about its function is lacking. Collagen XXIII has structural features in common with collagen XIII and may therefore have a similar function (Hagg et al. 2001; Koch et al. 2006). Type XIII collagen is expressed in almost all connective tissue-producing cells (Snellman et al. 2000) and is important for the regulation of adhesion of mesenchymal cells to the surrounding ECM and neighbouring cells, thereby facilitating transmembrane signalling (Peltonen et al. 1999). In addition, data suggest the involvement of collagen XIII in numerous differentiation and maturation processes associated with inflammation and vasculogenesis (Heikkinen et al. 2012). Positive correlations between inflammatory markers and weight regain after energy restriction have been reported (Kong et al. 2013). Together with our findings, it suggests that people with a G/A genotype for *COL23A1* are predisposed to regaining weight due to differences in ECM biosynthesis and inflammatory profile.

Studies of the skin have revealed that collagen XXIII can bind as a ligand to integrin α_2_β_1_, which directly interacts with the LAMB1-containing trimer laminin111 (Veit et al. 2011). In fact, FBLN5 also binds to β-integrin and, in the mouse, it was observed that FBLN5 competes with FN for binding to integrin-β1 (Schluterman et al. 2010). Altogether, four of the five genes identified here seem to interact with integrin-β, which suggests that integrins may play a key role in the influence of the ECM on weight regain. Moreover, a sex-specific preference for interaction with integrin-β1 could explain our result that in men variation in the *FBLN5* gene and in women variation in the *FN1* gene are associated with weight regain after weight loss.

Among females, we observed that an A/A genotype in the *FN1* gene is associated with weight regain. Our finding is in line with that of Mutch et al. (2011) who demonstrated that *FN1* was upregulated in subjects regaining weight after weight loss, while the opposite was observed in subjects maintaining their lost weight. Fibronectin, an important component of the ECM, functions both as regulator of various cellular processes and as scaffolding protein maintaining and directing tissue organisation and ECM composition (To and Midwood 2011). In morbid obese subjects, elevated plasma levels of fibronectin are found, and when these subjects lose weight, fibronectin levels normalise (Andersen et al. 1987). During adipocyte differentiation, fibronectin levels decrease (Antras et al. 1989) and culturing preadipocytes on fibronectin-coated dishes prevent adipocyte differentiation (Kamiya et al. 2002). In this respect, fibronectin seems to influence the differentiation potential of preadipocytes. Although weight regain after weight loss is supposed to be mainly due to renewed fat accumulation in mature adipocytes (Kolehmainen et al. 2008; Mariman 2011, 2012), to some extent differentiation of preadipocytes can also occur (Rossmeislova et al. 2013). Therefore, the predisposition to weight regain after weight loss mediated by the detected genetic variation in ECM genes may in part result from interference with volume changes in adipocytes and in part from influencing differentiation of preadipocytes.

Although DiOGenes is one of the largest weight loss-maintenance studies, a limitation of the current study is the relatively small number of participants. Because of this limited number, we did not take the variation in the maintenance diet into account. Genotype analysis of the total cohort of 469 subjects did not lead to significant results. Also, no functional analysis of the detected genetic variation was performed. Yet, this study produces leads for understanding the role of the ECM and its genetic variation in weight regain after weight loss, which should be confirmed and can be extended in larger cohorts.

In conclusion, we have investigated the role of genetic variation in ECM genes in regard to weight regain. Our results illustrate an involvement of variants of the *POSTN*, *LAMB1*, *COL23A1*, and *FBLN5* genes for males and of the *FN1* gene for females in the risk of weight regain after weight loss. Influence on weight regulation may come from the level of cell stress generated between the ECM and the adipocyte during weight loss, but also from modifying the differentiation of preadipocytes. Further research is required to confirm our findings in a larger cohort and to bring more clarity in the mechanism of weight regain after weight loss.

## Electronic supplementary material

Supplementary material 1 (PDF 158 kb)

Supplementary material 2 (PDF 1154 kb)
