# Supplementary material for: Variation in extracellular matrix genes is associated with weight regain after weight loss in a sex-specific manner
Source: Genes Nutr. 2015 Nov 19;10(6):56. doi: 10.1007/s12263-015-0506-y (PMC4653119; doi:10.1007/s12263-015-0506-y)
Supplement: Supplementary file 1 — Supplementary material 1 (PDF 158 kb) [file 12263_2015_506_MOESM1_ESM.pdf]

Supplement table 1: list of 124 candidate genes related to extracellular matrix

gene	count_dbSNPs	count_genotyped!	chr	start	end
ACAN	1750	33	15	89346674	89418585
ADAM11	572	6	17	42836399	42859214
ADAM12	6981	167	10	1,28E+08	1,28E+08
ADAM15	532	9	1	1,55E+08	1,55E+08
ADAM17	1278	11	2	9628615	9695921
ADAM19	3290	43	5	1,57E+08	1,57E+08
ADAM22	3882	43	7	87563458	87832204
ADAMTS1	509	15	21	28208606	28217728
ADAMTS4	489	4	1	1,61E+08	1,61E+08
ADAMTS5	1147	19	21	28290231	28338832
AZGP1	489	3	7	99564343	99573780
BGN	463	8	X	1,53E+08	1,53E+08
BMP1	1366	15	8	22022249	22069839
CALR	498	4	19	13049392	13055303
CCDC80	1058	17	3	1,12E+08	1,12E+08
CHI3L1	565	13	1	2,03E+08	2,03E+08
CILP	653	9	15	65488337	65503826
CNTNAP1	554	2	17	40834631	40851832
COL11A1	4295	53	1	1,03E+08	1,04E+08
COL12A1	2055	22	6	75794042	75915767
COL14A1	4927	69	8	1,21E+08	1,21E+08
COL15A1	2501	39	9	1,02E+08	1,02E+08
COL18A1	2951	37	21	46825052	46933634
COL1A1	1111	6	17	48260650	48278993
COL1A2	1257	25	7	94023873	94060544
COL23A1	7017	135	5	1,78E+08	1,78E+08
COL2A1	1089	25	12	48366748	48398269
COL3A1	975	20	2	1,9E+08	1,9E+08
COL4A1	3541	89	13	1,11E+08	1,11E+08
COL4A2	4826	84	13	1,11E+08	1,11E+08
COL4A3	2897	67	2	2,28E+08	2,28E+08
COL4A5	2927	6	X	1,08E+08	1,08E+08
COL5A1	4749	79	9	1,38E+08	1,38E+08
COL5A2	2410	10	2	1,9E+08	1,9E+08
COL5A3	1512	17	19	10070237	10121147
COL6A1	1091	6	21	47401651	47424964
COL6A2	1596	17	21	47518011	47552763
COL6A3	2292	42	2	2,38E+08	2,38E+08
CSPG4	895	5	15	75966663	76005189
CTGF	321	7	6	1,32E+08	1,32E+08
DAG1	1125	6	3	49506146	49573048
DCN	837	10	12	91539025	91576900
DPT	1056	23	1	1,69E+08	1,69E+08
ECM1	439	2	1	1,5E+08	1,5E+08
ECM2	828	6	9	95256365	95298937
EFEMP1	1133	15	2	56093102	56151274
EFEMP2	543	2	11	65633912	65641063

ELN	885	10	7	73442119	73484237
EMILIN1	452	2	2	27301435	27309271
EMILIN2	1738	26	18	2847028	2915991
FBLN1	2283	50	22	45898118	45997015
FBLN2	2283	42	3	13573824	13679922
FBLN5	1620	42	14	92335756	92414331
FBN1	3921	33	15	48700503	48938046
FKBP9	1160	3	7	32997017	33046543
FMOD	606	8	1	2,03E+08	2,03E+08
FN1	1616	16	2	2,16E+08	2,16E+08
GPC1	960	14	2	2,41E+08	2,41E+08
ITGAM	1271	10	16	31271311	31344213
ITGAV	1975	14	2	1,87E+08	1,88E+08
ITGB1	1998	23	10	33189247	33294720
ITGB2	1409	29	21	46305868	46351904
ITGB5	2300	42	3	1,24E+08	1,25E+08
LAMA2	9937	125	6	1,29E+08	1,3E+08
LAMA4	2667	53	6	1,12E+08	1,13E+08
LAMB1	1809	25	7	1,08E+08	1,08E+08
LAMB2	532	3	3	49158547	49170551
LAMC1	2218	18	1	1,83E+08	1,83E+08
LGALS1	403	5	22	38071615	38075813
LGALS3BP	561	9	17	76967320	76976191
LOX	540	6	5	1,21E+08	1,21E+08
LOXL1	723	16	15	74218330	74244478
LUM	500	5	12	91496406	91505608
MATN2	2763	71	8	98881068	99048944
MATN3	548	6	2	20191872	20212455
MATN4	725	9	20	43922085	43937169
MFAP4	359	2	17	19286755	19290553
MMP1	549	11	11	1,03E+08	1,03E+08
MMP10	579	15	11	1,03E+08	1,03E+08
MMP14	614	10	14	23305766	23318236
MMP19	464	1	12	56229217	56236750
MMP2	2624	55	16	55423612	55540603
MMP9	685	6	20	44637547	44645200
NID1	1900	28	1	2,36E+08	2,36E+08
NID2	1766	43	14	52471521	52535712
NPNT	1984	21	4	1,07E+08	1,07E+08
OGN	517	2	9	95146249	95166978
P4HA1	1713	5	10	74766975	74856732
P4HB	824	1	17	79801035	79818570
PCOLCE	424	2	7	1E+08	1E+08
PDIA3	690	2	15	44038590	44065477
PDIA4	877	6	7	1,49E+08	1,49E+08
PDIA6	1696	23	2	10923517	10978103
PLOD1	1131	8	1	11994262	12035595
PLOD2	1653	13	3	1,46E+08	1,46E+08
PLOD3	678	6	7	1,01E+08	1,01E+08
POSTN	909	11	13	38136720	38172981

PPIA	720	1	7	44836279	44864163
PPIB	414	2	15	64448011	64455404
PRELP	689	0	1	2,03E+08	2,03E+08
PRG4	682	8	1	1,86E+08	1,86E+08
PRSS3P1	545	0	7	1,42E+08	1,42E+08
SERPINA12	993	27	14	94953611	94984181
SERPINE1	593	7	7	1,01E+08	1,01E+08
SERPINH1	528	10	11	75273101	75283828
SPARC	730	15	5	1,51E+08	1,51E+08
SPARCL1	1206	22	4	88394487	88452213
SPON1	4807	75	11	13983914	14289646
SPON2	1012	10	4	1160720	1202750
TGFBI	905	12	5	1,35E+08	1,35E+08
TGFBR3	3798	80	1	92145902	92371892
THBS1	654	9	15	39873280	39891667
THBS2	1244	18	6	1,7E+08	1,7E+08
THBS3	492	2	1	1,55E+08	1,55E+08
TIMP1	269	3	X	47441712	47446188
TIMP2	1731	22	17	76849059	76921469
TIMP3	1298	35	22	33197687	33259030
TIMP4	389	4	3	12194551	12200851
TLL1	4402	45	4	1,67E+08	1,67E+08
TLL2	2629	49	10	98124363	98273675
TNC	2085	47	9	1,18E+08	1,18E+08
TNN	1826	20	1	1,75E+08	1,75E+08
TNXB	1307	17	6	32008931	32083111
VCAN	2136	43	5	82767284	82878122
