# Supplementary material for: Variation in extracellular matrix genes is associated with weight regain after weight loss in a sex-specific manner
Source: Genes Nutr. 2015 Nov 19;10(6):56. doi: 10.1007/s12263-015-0506-y (PMC4653119; doi:10.1007/s12263-015-0506-y)
Supplement: Supplementary file 2 — Supplementary material 2 (PDF 1154 kb) [file 12263_2015_506_MOESM2_ESM.pdf]

Supplement table 2: list of 2903 single nucleotide polymorphisms for which genotype data was retrieved.

snp	gene	allele	snp_pos
rs684	ITGB2	CT	46306161
rs1085	COL11A1	AC	103356168
rs1164	EMILIN2	CT	2917152
rs2429	COL14A1	GT	121384163
rs3522	LOXL1	CT	74244344
rs4415	FBLN1	AG	45971880
rs4416	FBLN1	AG	45972017
rs4572	TGFBI	GT	135399452
rs4625	DAG1	AG	49572140
rs4715	BMP1	AC	22021037
rs4799	PDIA6	CT	10933249
rs4807	PDIA6	CT	10930903
rs7242	SERPINE1	GT	100781445
rs7381	FBLN1	CT	45996782
rs8089	THBS2	AC	169617726
rs8111	TNXB	CT	32083175
rs8992	ADAMTS1	AG	28208954
rs9254	COL6A1	AG	47424346
rs9862	TIMP3	CT	33253280
rs11369	SPON1	AG	14288096
rs12140	ADAMTS1	CT	28209395
rs13294	ECM1	AG	150484987
rs13615	ADAMTS1	AG	28209667
rs13652	FN1	CT	216249587
rs13817	EFEMP2	AG	65628334
rs17576	MMP9	AG	44640225
rs20563	LAMC1	AG	183085755
rs25659	LAMB1	AG	107626770
rs28296	VCAN	CT	82760017
rs33598	VCAN	CT	82775634
rs33599	VCAN	AG	82774904
rs33604	VCAN	AG	82770586
rs42518	COL1A2	CT	94038934
rs42523	COL1A2	CT	94040999
rs42530	COL1A2	AG	94052495
rs72509	COL4A1	GT	110903187
rs80272	TIMP3	CT	33224779
rs80610	FBLN1	AG	45971291
rs130274	TIMP3	CT	33204334
rs130293	TIMP3	AG	33221939
rs130301	TIMP3	GT	33234199
rs130559	TIMP3	CT	33188536
rs130561	TIMP3	AC	33191373
rs134821	FBLN1	CT	45970029

rs135026	TIMP3	CT	33212769
rs135028	TIMP3	CT	33228070
rs135029	TIMP3	AG	33240290
rs135153	TIMP3	CT	33191491
rs136723	FBLN1	AG	45916679
rs136739	FBLN1	AG	45928147
rs136740	FBLN1	AG	45928324
rs136753	FBLN1	CT	45936121
rs136756	FBLN1	AG	45937061
rs137484	TIMP3	CT	33251279
rs137487	TIMP3	AG	33259104
rs137489	TIMP3	CT	33262935
rs151058	ADAMTS5	CT	28311572
rs151779	MMP2	CT	55447824
rs160277	VCAN	GT	82837631
rs160283	VCAN	AG	82859485
rs160380	VCAN	AG	82810714
rs162496	ADAMTS5	AG	28306575
rs162512	ADAMTS5	CT	28284577
rs162524	ADAMTS5	CT	28282383
rs169496	TNXB	CT	32052983
rs170962	ITGB2	CT	46295995
rs173686	VCAN	AG	82811500
rs183112	MMP2	AG	55527682
rs185493	COL23A1	CT	177991258
rs185819	TNXB	CT	32050067
rs188703	VCAN	AG	82834299
rs190145	ADAMTS1	CT	28203478
rs204899	TNXB	CT	32057627
rs226794	ADAMTS5	AG	28302355
rs229032	ADAMTS1	AG	28200449
rs229053	ADAMTS5	AC	28316268
rs229054	ADAMTS5	AG	28316816
rs229078	ADAMTS5	GT	28296135
rs229079	ADAMTS5	AG	28298049
rs229100	ADAMTS1	CT	28223092
rs233598	ADAMTS5	GT	28330838
rs233896	ADAMTS5	GT	28324568
rs235305	ITGB2	AG	46298869
rs235325	ITGB2	AG	46311264
rs235326	ITGB2	AG	46311813
rs235329	ITGB2	GT	46314440
rs240366	COL12A1	CT	75915356
rs240369	COL12A1	AG	75919484
rs240721	COL12A1	AG	75879135
rs240724	COL12A1	AG	75852488
rs240734	COL12A1	AG	75847036
rs240736	COL12A1	AG	75848181

rs241905	TIMP3	CT	33266865
rs242076	TIMP3	AG	33229830
rs242078	TIMP3	AG	33228692
rs242089	TIMP3	AG	33213319
rs243834	MMP2	AG	55536687
rs243840	MMP2	AG	55528159
rs243842	MMP2	CT	55527422
rs243866	MMP2	AG	55511537
rs251124	VCAN	CT	82805424
rs256459	VCAN	CT	82775906
rs262020	COL23A1	CT	177964317
rs262023	COL23A1	AG	178025087
rs262037	COL23A1	CT	177990886
rs262065	COL23A1	AC	177999447
rs265332	LAMA2	AG	129551233
rs265365	LAMA2	AG	129556965
rs265371	LAMA2	CT	129600523
rs265387	LAMA2	AG	129564944
rs265395	LAMA2	AG	129594759
rs265402	LAMA2	GT	129578216
rs284142	TGFBR3	CT	92256626
rs284169	TGFBR3	AG	92213442
rs284170	TGFBR3	CT	92214628
rs284175	TGFBR3	AG	92222275
rs284176	TGFBR3	AG	92223976
rs284180	TGFBR3	AC	92227848
rs284198	TGFBR3	GT	92238167
rs284202	TGFBR3	AG	92241033
rs284877	TGFBR3	CT	92173796
rs308362	VCAN	AG	82823791
rs308363	VCAN	AG	82838532
rs309556	VCAN	CT	82840259
rs309561	VCAN	AG	82830308
rs309581	VCAN	CT	82871546
rs309596	VCAN	CT	82844728
rs309599	VCAN	AG	82881455
rs310504	VCAN	CT	82820278
rs310517	VCAN	GT	82814521
rs331380	ECM2	CT	95256519
rs369982	COL1A2	AG	94048482
rs377258	LAMA2	GT	129533324
rs387298	COL4A2	AG	111159628
rs390955	ADAMTS1	AC	28218457
rs392840	ADAMTS1	CT	28218381
rs400218	COL1A2	AG	94056006
rs400532	COL4A2	AC	111154631
rs406174	COL4A2	AG	111155363
rs411717	COL1A2	CT	94033031

rs413756	COL4A2	CT	111143755
rs421587	COL1A2	AG	94043274
rs428815	TGFBR3	CT	92260920
rs441051	COL1A2	CT	94054000
rs445348	COL4A2	AG	111158874
rs467691	ADAMTS5	GT	28286590
rs468276	ADAMTS1	CT	28203651
rs470168	MMP10	AG	102641382
rs470215	MMP1	CT	102661099
rs470215	MMP10	CT	102661099
rs470504	MMP1	CT	102658689
rs470504	MMP10	CT	102658689
rs470747	MMP1	AG	102661595
rs471972	DPT	GT	168701085
rs472238	COL23A1	CT	177968768
rs489777	COL23A1	CT	177960872
rs492560	COL4A1	CT	110919976
rs497888	COL4A1	CT	110916553
rs498186	MMP1	AC	102669645
rs501630	EFEMP2	AG	65637273
rs504596	COL23A1	AG	177974127
rs506739	EMILIN2	AC	2856536
rs515242	COL23A1	AG	177963524
rs519806	MMP1	AG	102675489
rs529041	COL4A1	CT	110860219
rs529949	DPT	CT	168691984
rs529998	VCAN	CT	82841764
rs535858	COL23A1	AC	177971352
rs536014	DPT	CT	168695477
rs538060	DPT	AC	168662302
rs539707	COL23A1	CT	177906494
rs540519	COL4A1	AG	110922195
rs545833	DPT	CT	168689940
rs547561	MMP10	CT	102643894
rs552125	COL4A1	CT	110938813
rs553780	COL23A1	CT	177961860
rs554152	COL12A1	CT	75789526
rs555417	COL23A1	AG	178010145
rs556674	COL12A1	AG	75811880
rs561168	COL4A1	CT	110884208
rs561437	COL4A1	CT	110936227
rs562992	COL4A1	GT	110908777
rs564195	COL23A1	CT	177965633
rs564487	COL23A1	GT	177977679
rs566806	DCN	AG	91559197
rs566974	COL23A1	AG	177985652
rs574430	COL4A1	AG	110852220
rs574482	DCN	AG	91538458

rs581753	DPT	AG	168679692
rs585219	COL23A1	CT	177936898
rs585433	SERPINH1	AG	75292601
rs587409	COL4A1	CT	110903507
rs595325	COL4A1	AC	110919205
rs596279	COL23A1	CT	177966921
rs599483	COL4A1	AG	110930364
rs606452	SERPINH1	AC	75276178
rs608585	SERPINH1	AG	75268704
rs609309	SERPINH1	AG	75287341
rs613116	COL4A1	AC	110808823
rs614664	ITGB5	AC	124486993
rs616008	COL4A1	CT	110878344
rs619152	COL4A1	AG	110939497
rs621209	ITGB5	AG	124494030
rs622432	COL23A1	CT	177910253
rs622486	COL4A1	AG	110848291
rs623561	EMILIN2	AG	2872586
rs625053	COL4A1	AC	110875942
rs625820	COL23A1	GT	177995909
rs626444	COL4A1	CT	110864396
rs627527	COL4A1	AG	110915135
rs629241	COL23A1	CT	177933945
rs630943	COL4A1	AG	110877046
rs631428	COL4A1	CT	110808681
rs631451	DPT	AG	168668005
rs635567	COL12A1	AC	75829646
rs637647	EMILIN2	CT	2881839
rs640649	SERPINH1	AG	75271851
rs642624	COL23A1	CT	177969010
rs642887	EMILIN2	AG	2874408
rs642926	EMILIN2	AG	2916054
rs644582	COL4A1	AG	110840028
rs648705	COL4A1	AC	110856153
rs652054	SERPINH1	AC	75275532
rs656533	COL4A1	AG	110793123
rs657635	FBN1	AC	48882966
rs668842	FBN1	CT	48891965
rs672949	COL4A1	CT	110879554
rs673482	SERPINH1	AC	75268603
rs674503	SERPINH1	CT	75268337
rs679153	EMILIN2	GT	2869916
rs679958	COL4A1	CT	110879007
rs680484	COL4A1	GT	110881085
rs682367	DPT	AG	168664450
rs684320	EMILIN2	AG	2851819
rs688074	COL23A1	AG	177965696
rs695103	VCAN	CT	82841476

rs713835	LGALS1	CT	38064650
rs713896	FBLN1	AG	45908895
rs715572	TIMP3	AG	33234931
rs716817	PDIA4	AG	148694939
rs716818	PDIA4	CT	148694971
rs717975	SERPINA12	CT	94944874
rs718041	TGFBI	CT	135370197
rs723556	TIMP1	AG	47432747
rs724617	FN1	AG	216282481
rs726063	FBLN5	CT	92373198
rs726328	ADAM22	CT	87739123
rs730179	TLL2	AG	98171325
rs732989	SPON1	AG	13978737
rs734566	TLL2	CT	98255314
rs734909	ADAM22	AC	87836113
rs736389	MATN4	CT	43946768
rs736846	DPT	AG	168695609
rs737088	TIMP3	CT	33187942
rs739215	FBLN1	AG	45967526
rs739270	FBLN1	AG	45943203
rs741198	FBLN5	CT	92367411
rs741212	DCN	AG	91574742
rs743257	MMP14	CT	23315331
rs743642	BGN	GT	152774221
rs743931	FBLN1	AG	45959540
rs746997	NID1	AG	236195181
rs748004	TLL2	GT	98158431
rs748289	ADAM12	CT	127837841
rs749167	NID1	CT	236177874
rs749259	COL5A3	CT	10113626
rs749425	COL23A1	CT	177751751
rs750449	FBLN1	CT	45894122
rs751181	COL23A1	GT	177776148
rs752283	COL23A1	CT	177682664
rs753833	FBLN2	CT	13606196
rs754423	NID2	CT	52527187
rs755251	FBN1	AG	48812020
rs756462	TGFBI	CT	135367945
rs756625	CILP	AG	65493309
rs757931	LAMB1	AC	107586518
rs760458	ITGB2	AC	46328917
rs760462	ITGB2	CT	46328099
rs762052	MMP14	AG	23308986
rs762438	COL6A2	CT	47536546
rs762625	CHI3L1	CT	203145301
rs762990	LGALS1	AG	38085475
rs763100	FBLN1	AC	45988571
rs763101	FBLN1	GT	45988612

rs810687	COL1A2	AG	94034848
rs837531	MMP2	CT	55494432
rs848792	ITGB5	CT	124574342
rs857587	PRG4	AG	186261061
rs857591	PRG4	GT	186257518
rs868589	ADAM12	CT	128002008
rs869776	COL15A1	CT	101810339
rs872328	ADAM12	CT	127710102
rs872587	COL4A2	AC	111015780
rs873957	ADAM12	AG	128003149
rs876043	FBLN5	AG	92357145
rs876084	COL14A1	CT	121101522
rs877336	MMP2	GT	55442987
rs879266	TLL2	AG	98263545
rs880295	ADAM11	CT	42848094
rs880633	CHI3L1	CT	203152801
rs881029	GPC1	CT	241403014
rs882727	COL15A1	AG	101730121
rs883873	TGFBR3	AG	92380302
rs889125	COL5A3	AC	10120571
rs889130	COL5A3	CT	10063445
rs890801	COL23A1	AG	177715291
rs890807	COL23A1	AG	177718389
rs890813	COL23A1	CT	177751488
rs893226	MMP2	GT	55502896
rs893260	MMP2	AG	55444847
rs893817	LOXL1	AG	74229065
rs893821	LOXL1	CT	74241235
rs894761	EMILIN2	AC	2856482
rs896056	FBLN2	AG	13583528
rs897189	MATN2	CT	98963817
rs897190	MATN2	AG	98945345
rs900030	SPON2	CT	1193832
rs901913	TGFBR3	GT	92293162
rs902370	LAMA2	AG	129605056
rs903352	COL4A1	AC	110917524
rs903354	COL4A1	GT	110916819
rs903355	COL4A1	AG	110952028
rs903355	COL4A2	AG	110952028
rs908810	FBLN2	AG	13674571
rs912946	COL4A2	AG	111147846
rs913746	COL4A2	AC	111016124
rs914991	COL15A1	AC	101834340
rs917055	COL2A1	AG	48385576
rs917205	ADAM22	AG	87717762
rs918542	COL5A3	CT	10071634
rs920686	CILP	AG	65511429
rs922146	TLL1	AG	166798354

rs924209	COL6A3	CT	238227536
rs927730	NID1	CT	236209883
rs928111	COL4A2	AC	111016886
rs929039	LGALS1	CT	38071511
rs929608	FBLN5	CT	92336775
rs930054	COL23A1	AC	177755433
rs933106	LAMA2	AG	129845907
rs937618	VCAN	CT	82782892
rs938605	ACAN	AG	89424581
rs938608	ACAN	GT	89398605
rs938613	ACAN	AG	89377773
rs938952	CILP	CT	65489128
rs939585	ACAN	CT	89378258
rs939590	ACAN	AG	89425779
rs944223	TNC	AG	117823775
rs944510	TNC	CT	117853022
rs944949	OGN	AG	95140613
rs945256	TNC	AG	117886024
rs946261	CHI3L1	CT	203157873
rs946262	CHI3L1	CT	203158229
rs946263	CHI3L1	AG	203165381
rs946615	NID2	CT	52481917
rs951958	ADAM19	AC	156889097
rs953288	TNC	AC	117805719
rs953386	COL4A1	CT	110943692
rs954326	COL2A1	GT	48394823
rs955293	COL14A1	AG	121080548
rs959537	COL14A1	GT	121253053
rs962066	VCAN	GT	82768214
rs964114	NID1	AG	236230900
rs964141	MATN2	AG	98962450
rs969139	LAMA4	CT	112446876
rs970547	COL12A1	CT	75797302
rs971394	NID2	AG	52522539
rs971402	LAMA4	AG	112487900
rs976240	COL15A1	AG	101797043
rs977795	MATN2	AG	98996027
rs981181	CCDC80	AC	112353238
rs985461	ADAM12	CT	127769545
rs985462	ADAM12	CT	127769598
rs990541	LAMA2	AG	129556517
rs994370	NPNT	CT	106923626
rs1000989	COL4A1	CT	110827303
rs1003349	MMP14	GT	23305663
rs1005913	MMP2	GT	55504521
rs1006666	ADAM12	CT	127854772
rs1007856	ITGB5	AG	124604858
rs1009002	ITGB1	AG	33236901

rs1010222	CALR	AG	13048608
rs1010831	COL14A1	AC	121257344
rs1012466	TNN	CT	175109884
rs1012808	SERPINA12	AG	94961086
rs1015081	ACAN	AG	89382608
rs1017723	ADAM12	CT	127746825
rs1017813	ITGB5	CT	124591985
rs1017957	TGFBR3	CT	92314131
rs1018139	TLL1	AG	166848001
rs1018643	COL4A2	AG	111096925
rs1018829	TNN	AG	175109722
rs1020235	ITGAV	CT	187447029
rs1025412	SPON1	AG	14246296
rs1025413	SPON1	CT	14247109
rs1028157	FBLN2	GT	13617049
rs1032255	TGFBR3	CT	92286940
rs1033813	COL15A1	CT	101698023
rs1034620	COL1A2	CT	94063415
rs1034762	COL2A1	AC	48389643
rs1036477	FBN1	AG	48914926
rs1039494	TLL1	AG	166941483
rs1041631	COL15A1	AG	101781858
rs1042630	ACAN	AG	89402051
rs1042704	MMP14	AG	23312594
rs1042917	COL6A2	AG	47545768
rs1050348	LAMA4	AG	112493872
rs1050813	SERPINE1	AG	100781615
rs1051069	NID2	AG	52478315
rs1051442	THBS1	CT	39887649
rs1051534	COL14A1	AG	121062077
rs1053312	COL6A1	AG	47423389
rs1053605	MMP2	CT	55519607
rs1056204	ADAM17	AC	9647091
rs1057285	TNN	CT	175126845
rs1058177	LAMC1	AG	183109171
rs1060545	TNC	AC	117821618
rs1061375	FBLN2	AG	13679203
rs1061947	COL1A1	AG	48262119
rs1063964	ADAM22	AG	87835469
rs1091811	ELN	AG	73491212
rs1091813	ELN	CT	73489736
rs1091814	ELN	AC	73489663
rs1107373	TNC	AG	117779933
rs1110163	COL23A1	AG	177761768
rs1120519	COL11A1	CT	103402184
rs1122869	COL3A1	AG	189829688
rs1123875	SPON1	CT	14253824
rs1126499	BGN	CT	152771509

rs1126823	ACAN	AG	89417238
rs1127648	CSPG4	AG	75967095
rs1130643	SPARCL1	CT	88412806
rs1131296	COL6A3	AG	238243292
rs1133219	COL4A1	AG	110813709
rs1134745	COL4A3	CT	228177567
rs1143678	ITGAM	CT	31343005
rs1143683	ITGAM	CT	31336888
rs1143914	COL4A1	AG	110867235
rs1150752	TNXB	CT	32064726
rs1150754	TNXB	CT	32050758
rs1151573	NID2	AG	52468641
rs1151578	NID2	GT	52477782
rs1151579	NID2	AG	52477835
rs1151582	NID2	CT	52482768
rs1152653	ADAM12	AG	127759334
rs1158471	COL14A1	CT	121277396
rs1158747	LAMA4	GT	112500650
rs1160798	LAMA4	CT	112438446
rs1170390	PLOD2	CT	145863779
rs1187056	ITGB1	AG	33260547
rs1187070	ITGB1	AC	33241138
rs1187078	ITGB1	AG	33249133
rs1187086	ITGB1	AG	33281942
rs1187095	ITGB1	CT	33266888
rs1192198	COL4A1	CT	110811578
rs1192201	COL4A1	AG	110806490
rs1192202	COL4A1	AC	110806524
rs1192524	TGFBR3	AG	92340684
rs1198873	PDIA6	CT	10924524
rs1208984	PLOD1	AG	11995176
rs1241163	COL11A1	AC	103355765
rs1241169	COL11A1	CT	103384610
rs1241182	COL11A1	AC	103348100
rs1250023	TNC	CT	117869930
rs1250105	SPON2	CT	1203265
rs1250126	SPON2	AG	1191042
rs1250248	FN1	AG	216287093
rs1250249	FN1	AG	216287276
rs1253682	NID2	CT	52463153
rs1265646	ITGB5	AG	124592700
rs1271449	TNC	CT	117852522
rs1273108	TNC	AG	117866424
rs1278278	ADAM12	AG	127753274
rs1278280	ADAM12	GT	127755544
rs1278282	ADAM12	AC	127756457
rs1278300	ADAM12	AG	127791184
rs1278302	ADAM12	AC	127798951

rs1278313	ADAM12	CT	127814489
rs1278315	ADAM12	CT	127815531
rs1278325	ADAM12	CT	127820978
rs1278327	ADAM12	AC	127822430
rs1278329	ADAM12	GT	127823151
rs1278331	ADAM12	CT	127823470
rs1278352	ADAM12	AG	127773376
rs1278377	ADAM12	AG	127869478
rs1293989	PRG4	AC	186267100
rs1320131	GPC1	CT	241411289
rs1323070	COL12A1	AG	75923605
rs1330351	TNC	AG	117840922
rs1330361	TNC	AG	117797394
rs1330362	TNC	CT	117779668
rs1330365	TNC	AG	117817683
rs1330368	TNC	AG	117821026
rs1342548	ITGB1	AG	33188102
rs1344733	EFEMP1	CT	56128027
rs1345319	MMP2	AG	55461759
rs1346787	EFEMP1	CT	56092612
rs1347653	MMP2	GT	55505040
rs1353681	TLL1	AG	167023255
rs1354306	TLL1	CT	166889511
rs1356168	COL5A2	AG	190018099
rs1360686	COL15A1	AG	101823327
rs1367228	EFEMP1	AC	56112440
rs1380439	ADAM12	CT	127768110
rs1382192	PDIA4	AG	148707278
rs1384364	TIMP2	AG	76852828
rs1390964	NID2	CT	52529923
rs1391334	TLL1	AC	167027707
rs1393851	TLL1	AG	166862885
rs1393855	TLL1	AG	166972657
rs1411456	TNC	AG	117803882
rs1412335	DPT	CT	168658880
rs1413294	COL15A1	AG	101725509
rs1413299	COL15A1	GT	101761241
rs1415363	COL11A1	AG	103552935
rs1420227	MMP2	CT	55465544
rs1420228	MMP2	AG	55485173
rs1422794	ADAM19	GT	156936549
rs1422795	ADAM19	CT	156936364
rs1423569	VCAN	CT	82803452
rs1426715	FBN1	AG	48741996
rs1427378	TIMP3	AG	33252041
rs1430197	EFEMP1	AG	56134827
rs1437245	MMP2	AC	55481549
rs1437248	MMP2	AG	55461198

rs1437799	FN1	CT	216282993
rs1448427	ITGAV	AG	187526672
rs1454713	MATN2	CT	99025466
rs1459709	ADAM12	AG	127872828
rs1459713	ADAM12	CT	127735594
rs1460060	TLL1	AC	166815429
rs1460062	TLL1	AG	166804284
rs1462359	SPARCL1	CT	88442417
rs1463034	COL11A1	CT	103455217
rs1463035	COL11A1	CT	103450196
rs1463039	COL11A1	CT	103361698
rs1468358	PLOD3	CT	100853173
rs1470425	COL4A1	AG	110819991
rs1470428	COL4A1	CT	110819854
rs1472057	LAMA2	CT	129653602
rs1474552	ITGB2	CT	46337290
rs1474868	PLOD1	CT	12044164
rs1475584	ADAMTS1	CT	28220730
rs1478604	THBS1	CT	39873321
rs1478804	LAMA2	AG	129429694
rs1478806	LAMA2	AG	129533619
rs1497077	NID2	CT	52491655
rs1497290	ADAMTS5	AG	28348083
rs1499966	ITGB5	AG	124585214
rs1503292	TLL1	AC	166867875
rs1503293	TLL1	CT	167010685
rs1503294	TLL1	AG	166905263
rs1516454	COL3A1	AG	189844000
rs1516797	ACAN	GT	89410314
rs1528640	SPON1	AG	14070828
rs1528648	SPON1	AG	14139686
rs1528654	SPON1	AG	14127493
rs1528657	SPON1	GT	13990544
rs1528658	SPON1	GT	13990280
rs1528667	SPON1	AG	14033524
rs1530169	LOXL1	CT	74237120
rs1536622	COL4A2	CT	111064526
rs1537504	COL15A1	AG	101829542
rs1537506	COL15A1	CT	101821169
rs1540923	PLOD1	AG	12032030
rs1543709	COL15A1	CT	101752089
rs1543803	TIMP3	GT	33265922
rs1544926	COL23A1	AG	177665342
rs1544928	COL23A1	AG	177710630
rs1545030	SPARC	CT	151067412
rs1545692	ADAM12	AG	127866255
rs1545751	COL23A1	AC	177980792
rs1548638	LAMB1	GT	107604818

rs1551679	ADAM12	GT	127854622
rs1553469	CNTNAP1	AC	40842762
rs1555890	TGFBR3	CT	92160886
rs1556122	COL4A2	CT	110985607
rs1556124	COL4A2	AG	110991189
rs1556220	TNC	CT	117812777
rs1556329	COL18A1	CT	46912386
rs1556791	DPT	CT	168700485
rs1558085	ADAM11	AG	42854208
rs1558670	MMP2	CT	55478046
rs1559144	ADAM19	AG	156881853
rs1562808	FBLN2	CT	13665148
rs1563389	COL14A1	CT	121301474
rs1563400	COL14A1	CT	121268269
rs1566128	NID2	AG	52514981
rs1566153	COL6A3	CT	238239441
rs1570831	NID1	AC	236153161
rs1609340	SPON1	CT	14124088
rs1609341	SPON1	GT	14124311
rs1621212	ADAM12	CT	127841282
rs1623958	PDIA6	GT	10980653
rs1624391	ADAM12	CT	127833685
rs1626087	PDIA6	CT	10963420
rs1635544	COL2A1	AG	48380031
rs1637490	ADAM22	AG	87628945
rs1637500	ADAM22	GT	87594188
rs1644733	COL5A3	CT	10129262
rs1663501	ADAMTS1	AG	28219307
rs1674925	ADAM12	GT	127850697
rs1674927	ADAM12	CT	127852395
rs1674934	ADAM12	CT	127857804
rs1676486	COL11A1	AG	103354138
rs1676509	COL11A1	CT	103375336
rs1676719	ADAM12	CT	127843169
rs1676731	ADAM12	CT	127848239
rs1676734	ADAM12	CT	127849558
rs1676741	ADAM12	AG	127851530
rs1678979	FBN1	CT	48896830
rs1678983	FBN1	CT	48930645
rs1686444	PDIA6	CT	10937409
rs1686447	PDIA6	CT	10935224
rs1686468	PDIA6	AG	10986626
rs1686484	PDIA6	AG	10970473
rs1686486	PDIA6	AG	10969564
rs1688886	ADAM22	AG	87603743
rs1688888	ADAM22	CT	87605618
rs1688890	ADAM22	CT	87606246
rs1707469	PLOD2	AC	145861305

rs1710278	ADAM12	AG	127738918
rs1710293	ADAM12	AC	127744829
rs1710309	ADAM12	CT	127733386
rs1710313	ADAM12	AC	127734513
rs1732164	LAMB1	AG	107649288
rs1734389	PDIA6	CT	10985364
rs1734394	PDIA6	CT	10986305
rs1734395	PDIA6	GT	10986902
rs1757095	TNC	CT	117848394
rs1757096	TNC	GT	117849641
rs1757106	TNC	GT	117845333
rs1790994	EMILIN2	CT	2913431
rs1793923	COL2A1	CT	48384122
rs1793949	COL2A1	AG	48371595
rs1793958	COL2A1	AG	48392433
rs1800222	COL1A2	CT	94030899
rs1804506	TGFBR3	CT	92148013
rs1805110	TGFBR3	AG	92327045
rs1805113	TGFBR3	AG	92177938
rs1805116	TGFBR3	CT	92149096
rs1816594	MMP2	CT	55489908
rs1819084	SPON1	AC	13996155
rs1826956	TLL1	CT	167019072
rs1830390	SPON1	AG	13996226
rs1846371	COL23A1	CT	177888966
rs1852755	SPON1	CT	13996686
rs1852757	SPON1	CT	13987892
rs1858822	COL1A2	AG	94032753
rs1861087	FBLN5	CT	92371353
rs1861320	MMP2	GT	55541040
rs1864658	SPON1	CT	14256444
rs1867667	VCAN	AG	82779013
rs1869609	MATN2	AG	98991221
rs1869610	MATN2	AC	98945834
rs1869612	MATN2	AG	98925477
rs1869831	COL14A1	AG	121292407
rs1870510	ADAM12	CT	127837577
rs1870881	SPARCL1	AG	88435789
rs1871054	ADAM12	CT	127782409
rs1873329	LAMA2	CT	129662357
rs1874573	COL6A3	AG	238301184
rs1876206	FBN1	CT	48900586
rs1878026	ADAM12	CT	127710314
rs1878027	ADAM12	CT	127698643
rs1878030	ADAM12	CT	127786509
rs1878172	FBLN2	AG	13642870
rs1878173	FBLN2	AG	13642946
rs1879529	ACAN	GT	89414295

rs1881512	SPON1	CT	14103801
rs1881516	SPON1	CT	13993498
rs1882435	COL4A3	AC	228102752
rs1885019	NID1	AG	236203911
rs1888221	TNC	AG	117778355
rs1889892	LAMA2	AG	129762310
rs1893152	EMILIN2	CT	2841862
rs1894681	LAMA4	AG	112504110
rs1913185	PLOD2	GT	145888457
rs1914037	COL3A1	AG	189845515
rs1919295	SPON1	CT	14070567
rs1919297	SPON1	GT	14089499
rs1919309	SPON1	CT	14029854
rs1922021	COL4A3	CT	228025756
rs1922022	COL4A3	CT	228030362
rs1923702	TLL2	GT	98115684
rs1926261	TGFBR3	CT	92162920
rs1927343	COL4A2	GT	111053959
rs1931002	CTGF	CT	132278482
rs1937090	TLL2	GT	98224776
rs1939008	MMP1	AG	102656423
rs1939008	MMP10	AG	102656423
rs1948307	LAMA2	CT	129638583
rs1950134	COL4A3	AG	228062952
rs1951007	SERPINA12	AG	94969696
rs1951017	SERPINA12	AG	94979283
rs1956280	NID2	CT	52542247
rs1968510	FN1	AG	216258251
rs1969539	SPON1	AG	14038621
rs1975919	COL11A1	AG	103403752
rs1977893	COL4A1	CT	110840860
rs1980977	COL18A1	AG	46816019
rs1980983	COL6A2	AG	47558994
rs1981316	COL4A1	AC	110851808
rs1982963	NID2	AG	52509101
rs1983931	COL4A2	AG	111118102
rs1990950	ADAM19	GT	156920756
rs1990951	ADAM19	CT	156920911
rs1992898	ITGAV	AG	187491140
rs1993392	COL14A1	AG	121240646
rs1995126	TLL1	AG	167014137
rs1996223	COL23A1	AG	177916502
rs1998207	SERPINA12	GT	94957714
rs2003241	TIMP2	CT	76885117
rs2010221	TNN	CT	175093867
rs2011616	EMILIN1	AG	27302561
rs2016518	FBLN1	AG	45983901
rs2018072	FBLN1	AG	45918491

rs2019185	CILP	AG	65500435
rs2021783	TNXB	CT	32044851
rs2022161	ADAM22	GT	87639968
rs2023533	ADAM22	AG	87634515
rs2025405	POSTN	CT	38170523
rs2025453	TLL2	AC	98231449
rs2026882	ITGB2	CT	46322853
rs2026886	COL18A1	AC	46830873
rs2027084	LAMC1	AG	183110801
rs2029137	PLOD2	AG	145798787
rs2029356	TGFBR3	CT	92209311
rs2029980	NID2	AC	52501235
rs2029981	NID2	CT	52501214
rs2031014	SPON1	CT	14051977
rs2032568	LAMA4	AG	112457471
rs2033204	COL23A1	CT	177707204
rs2033316	EFEMP1	AG	56140531
rs2033467	SPARC	CT	151039626
rs2034843	COL14A1	AG	121343774
rs2035265	ITGB5	GT	124488610
rs2038498	SERPINA12	CT	94952404
rs2041009	ADAM22	AG	87763113
rs2045819	COL11A1	CT	103400314
rs2046736	TGFBR3	CT	92175008
rs2050601	COL11A1	AG	103498029
rs2051616	FBLN1	AG	45963523
rs2051649	LAMA4	AG	112490531
rs2052204	FBLN5	AG	92330819
rs2061052	MATN2	CT	99053025
rs2061054	MATN2	AG	98994851
rs2061516	TLL1	CT	166801301
rs2061705	COL11A1	ACGT	103441814
rs2067986	COL15A1	CT	101816399
rs2070682	SERPINE1	CT	100777267
rs2070739	COL2A1	CT	48367976
rs2070871	P4HB	AG	79805134
rs2070947	ITGB2	AG	46340843
rs2071232	MMP1	CT	102665669
rs2071295	TNXB	CT	32038700
rs2071307	ELN	AG	73470714
rs2071358	COL2A1	GT	48366449
rs2071869	FBLN1	AG	45972551
rs2071870	FBLN1	AG	45972610
rs2071871	FBLN1	AG	45996110
rs2071938	FBLN1	CT	45931262
rs2071947	TIMP3	CT	33246032
rs2072020	LAMA4	AG	112460648
rs2072024	LAMA4	CT	112558763

rs2072026	LAMA4	CT	112463076
rs2072038	TNN	CT	175105778
rs2072040	TNN	AG	175096333
rs2072041	TNN	CT	175092840
rs2072168	PLOD3	GT	100861870
rs2072209	LAMB1	AG	107592198
rs2072793	MATN4	AG	43941226
rs2073479	BGN	CT	152772473
rs2073622	NID2	AG	52500178
rs2073625	NID2	CT	52488688
rs2073711	CILP	AG	65494212
rs2074685	PLOD3	CT	100862264
rs2074811	TGFBI	CT	135391947
rs2075664	COL15A1	AC	101749498
rs2075666	COL15A1	CT	101798002
rs2076023	MATN4	AG	43929659
rs2076722	ITGB5	CT	124547871
rs2077911	SERPINA12	CT	94983224
rs2078114	ADAM12	CT	127776336
rs2083425	ITGB5	GT	124496888
rs2085144	MATN2	AG	98953076
rs2093558	TLL2	AC	98140048
rs2101575	LAMA2	AC	129616516
rs2101919	NID2	CT	52520368
rs2107331	TGFBI	AC	135377349
rs2107349	AZGP1	CT	99558440
rs2118181	FBN1	CT	48915884
rs2124471	COL14A1	AC	121179997
rs2126642	COL11A1	AG	103405793
rs2126643	COL11A1	CT	103404384
rs2129975	TGFBR3	GT	92282080
rs2131939	COL4A1	AG	110831837
rs2141831	COL4A3	CT	228087689
rs2147060	OGN	CT	95160477
rs2150101	TNC	AC	117812891
rs2151616	COL15A1	AG	101729103
rs2153875	ITGB1	AC	33190567
rs2154203	TLL2	AG	98212575
rs2156507	NPNT	CT	106913997
rs2158836	LAMB1	AG	107580839
rs2160079	FBLN5	AC	92374405
rs2160080	FBLN5	GT	92374487
rs2161396	ADAM19	AC	156868224
rs2162765	COL23A1	CT	177744380
rs2164072	ITGB2	AC	46301008
rs2165241	LOXL1	CT	74222202
rs2169076	ADAM12	CT	127849127
rs2169501	SPARCL1	AG	88461636

rs2176621	FBLN2	GT	13665410
rs2178242	SPON1	CT	14097139
rs2183589	COL18A1	CT	46821491
rs2197787	ITGAV	GT	187490371
rs2198747	COL14A1	CT	121310102
rs2198751	COL14A1	CT	121180667
rs2204862	COL4A3	AG	228052600
rs2207216	DPT	AC	168694212
rs2213842	LAMA4	AG	112423810
rs2227275	MATN4	CT	43926573
rs2227631	SERPINE1	AG	100769538
rs2227672	SERPINE1	GT	100775686
rs2227684	SERPINE1	AG	100776931
rs2228331	GPC1	AG	241405528
rs2229783	COL11A1	AG	103352451
rs2235133	ITGB2	CT	46321172
rs2236240	SERPINA12	CT	94960876
rs2236241	SERPINA12	AG	94960149
rs2236409	TNC	AC	117821746
rs2236451	COL18A1	AG	46876083
rs2236459	COL18A1	AG	46897079
rs2236462	COL18A1	CT	46897221
rs2236470	COL18A1	AG	46903021
rs2236472	COL18A1	CT	46903412
rs2236475	COL18A1	AG	46915042
rs2236479	COL18A1	AG	46919132
rs2236483	COL18A1	CT	46926054
rs2236490	COL6A2	AC	47512330
rs2237238	LAMA4	AC	112514717
rs2237244	LAMA4	CT	112524506
rs2237247	LAMA4	AG	112529849
rs2237248	LAMA4	AG	112538465
rs2237538	ADAM22	AG	87625967
rs2237541	ADAM22	CT	87731692
rs2237686	LAMB1	CT	107575162
rs2237687	LAMB1	CT	107585473
rs2237690	LAMB1	CT	107586254
rs2237701	LAMB1	AG	107622150
rs2238810	FBLN1	AG	45912376
rs2239400	FBLN1	CT	45936264
rs2239689	TNXB	AG	32030284
rs2239844	FBLN1	AG	45927112
rs2241579	COL23A1	GT	177669413
rs2241581	COL23A1	CT	177668882
rs2242278	SPON2	AG	1165516
rs2242295	MMP19	AG	56232176
rs2244008	LAMA2	AG	129813053
rs2244554	MATN2	AG	98963242

rs2246416	FBLN5	AG	92390620
rs2248014	MATN2	CT	99030280
rs2248052	FBLN5	CT	92352114
rs2249956	LAMB1	CT	107641616
rs2250509	CHI3L1	AG	203138970
rs2254320	FBLN5	AG	92371141
rs2255317	MATN2	CT	99044528
rs2255464	MATN2	AC	99045978
rs2256367	MATN2	CT	98930457
rs2266862	BGN	CT	152764518
rs2267184	TIMP3	CT	33258050
rs2267994	FBLN5	AG	92356946
rs2268578	LUM	AG	91501198
rs2269426	TNXB	AG	32076499
rs2269429	TNXB	CT	32029183
rs2269646	LAMA4	CT	112511852
rs2271649	ADAM12	AG	127702393
rs2272023	ACAN	AC	89391160
rs2273289	PLOD1	CT	12018290
rs2273429	NID2	AG	52494072
rs2273431	NID2	CT	52496407
rs2273779	PRG4	CT	186273994
rs2274545	COL4A2	AC	111145310
rs2276108	MMP10	CT	102647536
rs2276338	ADAM17	CT	9645789
rs2276454	COL2A1	AG	48376291
rs2276747	FBLN2	AG	13649762
rs2277027	ADAM19	AC	156932376
rs2277084	LAMA4	CT	112476210
rs2277814	COL6A1	AG	47409503
rs2277887	EFEMP1	CT	56145541
rs2277968	COL5A3	CT	10071669
rs2278230	COL23A1	CT	177690539
rs2279119	MATN2	CT	99046298
rs2279120	MATN2	CT	99046040
rs2279455	TGFBR3	CT	92185185
rs2279531	CCDC80	CT	112356770
rs2279723	TLL1	AC	167022359
rs2280261	GPC1	CT	241408125
rs2280305	FBLN2	CT	13660809
rs2280465	ACAN	AG	89417629
rs2281973	COL4A2	CT	111130674
rs2282117	COL18A1	GT	46889666
rs2282636	EMILIN2	CT	2921777
rs2282791	TGFBI	GT	135377730
rs2282854	LAMA4	CT	112531660
rs2283017	AZGP1	AG	99574758
rs2283657	FBLN1	AG	45910152

rs2283663	FBLN1	AG	45941476
rs2283885	TIMP3	AG	33248902
rs2284337	FBLN5	AG	92340722
rs2284339	FBLN5	AG	92340901
rs2284340	FBLN5	CT	92389047
rs2284341	FBLN5	AC	92389114
rs2285182	FBLN1	CT	45938846
rs2286024	COL2A1	AG	48360620
rs2286025	COL2A1	CT	48359984
rs2287749	ADAM19	CT	156918850
rs2287802	COL5A3	AG	10112688
rs2287807	COL5A3	CT	10097667
rs2287808	COL5A3	CT	10097593
rs2287810	COL5A3	CT	10087200
rs2287926	VCAN	AG	82815408
rs2288810	SPARC	AG	151045061
rs2289136	FBN1	AG	48944272
rs2289200	FN1	AC	216232978
rs2290192	FBLN2	CT	13671758
rs2290471	MATN2	AG	98943545
rs2290472	MATN2	CT	98943598
rs2290519	COL14A1	CT	121244066
rs2290523	COL14A1	AG	121354851
rs2290524	COL14A1	AG	121354962
rs2290673	VCAN	AG	82850631
rs2291081	ITGB5	AG	124485235
rs2291084	ITGB5	AG	124488185
rs2291090	ITGB5	AC	124515646
rs2291794	COL6A3	AG	238322579
rs2291795	COL6A3	CT	238253065
rs2292013	VCAN	AC	82792625
rs2292082	TLL1	AG	166996347
rs2292305	THBS1	AG	39880822
rs2292832	GPC1	CT	241395503
rs2296620	TGFBR3	AG	92174563
rs2296621	TGFBR3	GT	92163786
rs2296845	COL4A2	GT	111100947
rs2296851	COL4A2	AG	111138255
rs2297179	TNC	GT	117810250
rs2297181	TNC	CT	117810036
rs2297602	COL15A1	CT	101788522
rs2297740	LAMA2	AG	129835814
rs2297741	LAMA2	AG	129785282
rs2299189	ADAM22	CT	87570995
rs2299204	ADAM22	CT	87794452
rs2299205	ADAM22	CT	87794707
rs2299418	COL1A2	AG	94032325
rs2300792	COL12A1	CT	75859635

rs2300795	COL12A1	AG	75825181
rs2301994	ELN	AG	73452263
rs2301995	ELN	AG	73452140
rs2302038	TGFBI	CT	135397018
rs2303099	COL5A3	GT	10116375
rs2303500	FBN1	AG	48739274
rs2303505	FBN1	GT	48712757
rs2303973	SPON1	AG	14264916
rs2303974	SPON1	AC	14264979
rs2303975	SPON1	AG	14276999
rs2304681	EMILIN1	AG	27315252
rs2304719	LOXL1	CT	74235500
rs2304721	LOXL1	AC	74240354
rs2305600	COL14A1	CT	121215991
rs2305605	COL14A1	AG	121259862
rs2305607	COL14A1	AG	121262775
rs2306887	TGFBR3	CT	92200218
rs2306942	LAMA2	AG	129635800
rs2311801	DAG1	AG	49568095
rs2326483	COL14A1	CT	121211847
rs2326761	LAMA2	AG	129541597
rs2333618	LAMC1	AG	183121028
rs2350310	FBLN2	CT	13637116
rs2366698	ADAM12	CT	127975335
rs2366705	ADAM12	CT	128058851
rs2372544	FN1	GT	216264270
rs2376280	COL11A1	AG	103568464
rs2376999	TIMP2	AG	76890864
rs2385057	NID1	AG	236207358
rs2391823	COL4A1	AG	110944795
rs2391824	COL4A1	AG	110961282
rs2391824	COL4A2	AG	110961282
rs2396463	COL4A3	AC	228044992
rs2397446	MMP2	CT	55455664
rs2403708	SPON1	AG	14072663
rs2408490	MMP1	CT	102672553
rs2430339	FBLN5	AG	92335304
rs2430340	FBLN5	AG	92337774
rs2430342	FBLN5	CT	92344244
rs2430343	FBLN5	CT	92345665
rs2430353	FBLN5	AC	92355117
rs2430363	FBLN5	AG	92365051
rs2430368	FBLN5	CT	92387541
rs2437089	LAMA2	CT	129704589
rs2437090	LAMA2	GT	129717246
rs2437094	LAMA2	AG	129715399
rs2437095	LAMA2	GT	129715289
rs2444860	MATN2	CT	99052895

rs2444871	MATN2	CT	98980663
rs2444876	MATN2	CT	98970756
rs2444882	MATN2	AG	98964771
rs2444889	MATN2	AG	98908198
rs2444891	MATN2	CT	99016723
rs2444895	MATN2	CT	99020949
rs2444896	MATN2	AC	99022009
rs2444907	MATN2	CT	98934748
rs2448011	LAMA2	CT	129592284
rs2451688	LAMA2	AG	129646565
rs2459113	ADAM12	CT	127745094
rs2474028	FBLN5	CT	92403660
rs2475193	ITGB1	AG	33272133
rs2480933	TNC	CT	117864609
rs2482077	TNC	CT	117851672
rs2482078	TNC	AG	117865376
rs2483675	LAMC1	CT	183118282
rs2488314	ITGB1	AG	33187464
rs2488320	ITGB1	CT	33198911
rs2488326	ITGB1	CT	33207497
rs2488336	ITGB1	CT	33236305
rs2489188	TGFBR3	CT	92332489
rs2498828	FBLN5	CT	92421275
rs2498829	FBLN5	AG	92375770
rs2498845	FBLN5	GT	92346858
rs2498852	FBLN5	AG	92416364
rs2498854	FBLN5	GT	92326415
rs2512024	MATN2	CT	98952826
rs2512028	MATN2	CT	98960813
rs2512033	MATN2	AG	98970479
rs2512040	MATN2	AG	98981529
rs2512043	MATN2	CT	98987918
rs2512046	MATN2	AG	98990399
rs2513812	MATN2	GT	99046942
rs2513837	MATN2	CT	98936151
rs2513843	MATN2	CT	98949206
rs2513848	MATN2	AG	98989071
rs2513851	MATN2	AG	99002078
rs2513852	MATN2	AC	99008727
rs2521205	COL1A2	GT	94046541
rs2521206	COL1A2	AG	94039187
rs2527923	AZGP1	CT	99567455
rs2528648	LAMB1	AG	107568599
rs2528650	LAMB1	CT	107600558
rs2528651	LAMB1	CT	107602624
rs2540707	MMP2	AC	55472551
rs2540718	MMP2	AG	55478556
rs2540726	MMP2	AG	55481111

rs2541312	VCAN	AG	82819185
rs2544408	NPNT	CT	106808040
rs2546629	COL23A1	AC	177883242
rs2546643	COL23A1	AG	177964444
rs2546645	COL23A1	CT	177947771
rs2553453	NPNT	AG	106808172
rs2571576	LAMA2	CT	129834224
rs2571577	LAMA2	CT	129798084
rs2571584	LAMA2	AG	129803485
rs2576561	MMP2	CT	55470974
rs2576562	MMP2	AG	55466774
rs2586488	COL1A1	AG	48265426
rs2586494	COL1A1	AC	48273155
rs2615977	COL11A1	AC	103452392
rs2616009	COL11A1	CT	103409558
rs2618164	THBS1	CT	39863872
rs2618516	SPON1	CT	14021639
rs2622839	COL11A1	CT	103476179
rs2622874	COL11A1	AC	103466917
rs2622878	COL11A1	GT	103439819
rs2630452	FBLN2	CT	13571228
rs2630453	FBLN2	CT	13571802
rs2645737	NID2	CT	52513311
rs2645743	NID2	CT	52511878
rs2645766	COL6A3	GT	238257896
rs2645772	COL6A3	AG	238276385
rs2645779	COL6A3	CT	238311612
rs2645782	COL6A3	AC	238317822
rs2645783	COL6A3	AG	238319712
rs2646254	COL6A3	CT	238267717
rs2646261	COL6A3	AG	238259387
rs2646262	COL6A3	CT	238258280
rs2646265	COL6A3	AG	238257013
rs2647691	COL23A1	CT	177886182
rs2652106	VCAN	GT	82794036
rs2672818	COL23A1	AG	177932674
rs2672824	COL23A1	CT	177887732
rs2672826	COL23A1	AG	177954516
rs2696247	COL1A1	AG	48269903
rs2696270	COL1A1	CT	48257812
rs2697823	SPON1	AG	14080925
rs2697827	SPON1	GT	14090461
rs2697828	SPON1	CT	14090922
rs2697832	SPON1	AG	14100888
rs2697846	SPON1	GT	14002819
rs2697852	SPON1	AG	14022014
rs2701034	LAMB1	AG	107613379
rs2701039	LAMB1	AG	107623542

rs2731343	FBLN2	CT	13564048
rs2731647	LOX	AG	121420426
rs2741523	MATN4	CT	43920446
rs2743275	MATN4	AG	43918591
rs2743312	MATN4	AG	43936155
rs2749881	NID2	AG	52515209
rs2770186	TGFBR3	CT	92378843
rs2799512	TGFBR3	CT	92309916
rs2799537	TGFBR3	AG	92293412
rs2799541	TGFBR3	AG	92355115
rs2810891	TGFBR3	AG	92154088
rs2810893	TGFBR3	CT	92144970
rs2830585	ADAMTS5	CT	28305212
rs2830592	ADAMTS5	AG	28345952
rs2830593	ADAMTS5	AG	28346179
rs2830594	ADAMTS5	AG	28348803
rs2838730	ITGB2	AG	46319492
rs2838733	ITGB2	CT	46323731
rs2838734	ITGB2	CT	46329740
rs2838735	ITGB2	CT	46335282
rs2838737	ITGB2	CT	46335580
rs2838738	ITGB2	AG	46344426
rs2838906	COL18A1	CT	46821907
rs2838913	COL18A1	AG	46837709
rs2838917	COL18A1	CT	46839904
rs2838922	COL18A1	CT	46846015
rs2838923	COL18A1	AG	46846944
rs2838927	COL18A1	CT	46848326
rs2838942	COL18A1	AG	46904833
rs2838950	COL18A1	CT	46926297
rs2839108	COL6A2	CT	47535248
rs2839110	COL6A2	AG	47538960
rs2839112	COL6A2	CT	47544388
rs2839116	COL6A2	AC	47548555
rs2839117	COL6A2	AG	47550754
rs2840140	COL23A1	CT	177892347
rs2856728	ELN	CT	73470782
rs2861580	TLL2	AG	98208287
rs2861583	TLL2	CT	98209983
rs2869678	SPARCL1	AG	88433880
rs2876021	LAMA2	AG	129509207
rs2876038	LAMA2	AG	129561742
rs2877453	MATN2	AC	99055222
rs2882676	ACAN	AC	89400339
rs2884289	PDIA6	AG	10959724
rs2887831	ITGAV	CT	187541810
rs2889529	TIMP2	AG	76897475
rs2895219	ECM2	CT	95300559

rs2899292	LGALS1	AG	38077718
rs2899417	FBN1	CT	48700103
rs2901990	TLL2	AG	98120109
rs2910111	COL23A1	CT	177793761
rs2910119	COL23A1	AG	177798295
rs2913756	COL23A1	CT	177725276
rs2913766	COL23A1	CT	177735777
rs2913774	COL23A1	CT	177739628
rs2913779	COL23A1	AG	177743772
rs2913784	COL23A1	AG	177749643
rs2913788	COL23A1	AC	177757764
rs2913810	COL23A1	AG	177779994
rs2913818	COL23A1	AG	177719916
rs2913844	COL23A1	CT	177692247
rs2913847	COL23A1	CT	177684682
rs2913851	COL23A1	CT	177675217
rs2913861	COL23A1	CT	177659075
rs2930121	ADAM12	AG	127874740
rs2930125	ADAM12	CT	127889157
rs2959646	MATN2	CT	98935189
rs2967890	CALR	AG	13040647
rs2973675	COL23A1	CT	177762425
rs2973677	COL23A1	AG	177761336
rs2973689	COL23A1	GT	177754580
rs2973696	COL23A1	AG	177748144
rs2973713	COL23A1	CT	177796121
rs2973719	COL23A1	CT	177801274
rs2973725	COL23A1	AG	177804032
rs2973737	COL23A1	AG	177815313
rs2973739	COL23A1	CT	177820134
rs2973741	COL23A1	AG	177821665
rs2973742	COL23A1	AG	177830769
rs2973765	COL23A1	CT	177707427
rs2973775	COL23A1	AG	177717723
rs2973776	COL23A1	CT	177718517
rs2973808	COL23A1	CT	177766580
rs2974754	CALR	CT	13061983
rs2979860	LOX	AG	121421864
rs2980042	BGN	AG	152755269
rs2980060	BGN	AC	152779715
rs2985159	POSTN	GT	38176489
rs3087657	PDIA3	AG	44063859
rs3088026	COL6A2	CT	47549613
rs3096169	VCAN	AG	82831964
rs3106796	COL3A1	AG	189849773
rs3109676	COL5A1	AG	137631198
rs3109678	COL5A1	AC	137625142
rs3109687	COL5A1	GT	137599992

rs3124297	COL5A1	AG	137616715
rs3124300	COL5A1	AG	137619862
rs3124929	COL5A1	CT	137667432
rs3124932	COL5A1	CT	137672012
rs3124934	COL5A1	CT	137678038
rs3128570	COL5A1	AG	137743309
rs3128575	COL5A1	CT	137736650
rs3128591	COL5A1	AG	137602119
rs3128597	COL5A1	AC	137620847
rs3128606	COL5A1	CT	137622923
rs3128615	COL5A1	AG	137641411
rs3128621	COL5A1	GT	137678306
rs3134954	TNXB	CT	32071893
rs3138189	DCN	CT	91565694
rs3138190	DCN	GT	91565527
rs3138196	DCN	AG	91564481
rs3138209	DCN	CT	91561165
rs3138287	DCN	AC	91540432
rs3210714	SPARC	CT	151041932
rs3213190	NID1	CT	236141174
rs3731663	MATN3	CT	20189089
rs3731667	MATN3	AC	20205197
rs3734031	ADAM19	AG	156940760
rs3734034	ADAM19	CT	156821217
rs3734287	LAMA4	CT	112454245
rs3735602	LAMB1	AG	107563586
rs3736487	COL3A1	AG	189855943
rs3736638	COL1A2	AC	94047266
rs3738441	TGFBR3	CT	92224067
rs3738525	NID1	AG	236144951
rs3738919	ITGAV	AC	187521260
rs3739799	COL15A1	CT	101784777
rs3742188	COL4A2	AG	111175244
rs3742207	COL4A1	GT	110818598
rs3742536	NID2	AG	52507429
rs3743110	PPIB	AG	64462463
rs3743398	ACAN	CT	89398407
rs3744477	ADAM11	AG	42827673
rs3744787	TIMP2	AG	76893671
rs3745013	EMILIN2	AG	2915219
rs3745582	COL5A3	AC	10077158
rs3745596	COL5A3	CT	10108929
rs3746972	ITGB2	AG	46327835
rs3749282	PLOD2	CT	145783440
rs3749782	TGFBI	AG	135398279
rs3750255	COL14A1	AG	121267736
rs3751488	MMP14	AG	23304094
rs3753019	COL18A1	CT	46924785

rs3753103	ADAM22	CT	87735798
rs3753579	PLOD1	AG	12038591
rs3753841	COL11A1	AG	103379918
rs3754021	TGFBR3	CT	92158751
rs3754237	NID1	AG	236177182
rs3755724	TIMP4	CT	12200906
rs3758314	COL15A1	CT	101702439
rs3758419	ADAM12	CT	127784884
rs3758420	ADAM12	CT	127784936
rs3760386	CNTNAP1	AG	40842084
rs3762096	TLL2	AG	98136250
rs3763392	SPARCL1	AC	88415884
rs3763468	COL1A2	AG	94021546
rs3765087	ADAM15	AC	155029253
rs3765160	COL3A1	AG	189868381
rs3766550	CHI3L1	GT	203141689
rs3766551	CHI3L1	CT	203141592
rs3768622	LAMC1	AG	183090011
rs3768781	ITGAV	AG	187481835
rs3772823	ITGB5	CT	124491517
rs3772832	ITGB5	AG	124515876
rs3772836	ITGB5	AC	124535635
rs3772839	ITGB5	CT	124539303
rs3772841	ITGB5	AC	124554130
rs3772845	ITGB5	CT	124560890
rs3773255	FBLN2	CT	13672732
rs3773271	FBLN2	CT	13646818
rs3773276	FBLN2	AG	13639259
rs3773281	FBLN2	CT	13630819
rs3773282	FBLN2	CT	13630307
rs3773283	FBLN2	CT	13626592
rs3773300	FBLN2	CT	13614411
rs3773364	TIMP4	AG	12189968
rs3775110	SPON2	AG	1162022
rs3777505	COL12A1	AC	75880623
rs3777510	COL12A1	CT	75902804
rs3777925	LAMA4	AG	112526101
rs3777926	LAMA4	CT	112526025
rs3777932	LAMA4	AG	112515430
rs3777947	LAMA4	CT	112433664
rs3778112	LAMA2	CT	129609591
rs3778119	LAMA2	AG	129590111
rs3778135	LAMA2	AC	129507909
rs3778141	LAMA2	CT	129466298
rs3780622	COL15A1	AG	101763633
rs3780873	ITGB1	AG	33213680
rs3781001	ADAM12	CT	127706028
rs3781013	ADAM12	AG	127764148

rs3781031	ADAM12	AG	127783034
rs3783101	COL4A1	AG	110871312
rs3783106	COL4A1	GT	110842153
rs3783113	COL4A1	CT	110834746
rs3783632	NID2	AC	52498111
rs3783937	FBLN5	CT	92407693
rs3784381	PPIB	GT	64449839
rs3784390	THBS1	AG	39871080
rs3784759	ACAN	CT	89383044
rs3785529	LGALS3BP	CT	76972960
rs3786701	COL5A3	CT	10124504
rs3788142	ITGB2	AG	46316640
rs3788145	ITGB2	CT	46317126
rs3788150	ITGB2	GT	46333802
rs3788151	ITGB2	CT	46337565
rs3788190	COL18A1	AG	46936958
rs3788654	FBLN1	AG	45971490
rs3788658	FBLN1	AG	45980298
rs3788662	FBLN1	CT	45981447
rs3788667	FBLN1	AC	45982830
rs3789236	ADAM22	CT	87820419
rs3789867	TNC	AC	117875688
rs3789868	TNC	CT	117875681
rs3789875	TNC	AC	117795288
rs3789945	TLL2	CT	98152036
rs3789950	TLL2	CT	98162791
rs3789955	TLL2	AG	98205419
rs3790995	COL6A3	AG	238259675
rs3790998	COL6A3	AC	238260610
rs3791679	EFEMP1	AG	56096892
rs3795965	MATN3	AG	20199089
rs3803229	COL4A2	AG	111134780
rs3803231	COL4A2	CT	111119342
rs3803237	COL4A2	AG	111117668
rs3804665	PLOD2	GT	145808330
rs3806256	ADAM15	CT	155035611
rs3810068	EMILIN2	CT	2846499
rs3810631	FBLN1	CT	45897997
rs3810632	FBLN1	CT	45898074
rs3810875	ADAM22	AC	87820986
rs3811149	COL5A1	AG	137708803
rs3811157	COL5A1	CT	137690953
rs3811158	COL5A1	CT	137690877
rs3811159	COL5A1	AG	137688657
rs3811624	GPC1	CT	241409456
rs3814221	TLL2	CT	98127590
rs3814474	LAMB1	CT	107644808
rs3814835	FBLN5	CT	92414380

rs3815746	COL5A3	CT	10079093
rs3816375	ITGAV	AG	187505486
rs3816411	FBLN2	CT	13677751
rs3816665	LAMA2	AG	129571330
rs3817004	TIMP4	AG	12195674
rs3820182	PRG4	AG	186290333
rs3820558	NID1	AC	236144754
rs3821536	ITGB5	CT	124483952
rs3822585	ADAM19	AG	156906986
rs3822696	ADAM19	CT	156957741
rs3824511	COL15A1	CT	101816552
rs3824637	ADAM12	AG	127740389
rs3825478	COL4A1	AG	110836472
rs3825481	COL4A1	CT	110806852
rs3825594	NID2	AG	52507206
rs3826638	EMILIN2	CT	2891847
rs3827848	COL5A1	AG	137707834
rs3827851	COL5A1	GT	137694369
rs3828134	COL6A3	AG	238260797
rs3828240	EFEMP1	CT	56125319
rs3828334	GPC1	AG	241390002
rs3829364	POSTN	CT	38174533
rs3847470	ADAM12	GT	128030244
rs3850459	COL11A1	CT	103517428
rs3853401	LOX	AG	121417687
rs3856465	PDIA6	AG	10916670
rs3857979	BMP1	CT	22055348
rs3858316	ADAM12	AG	127927283
rs3858320	ADAM12	AG	127978792
rs3863065	ITGB5	GT	124550191
rs3866330	DAG1	CT	49582994
rs3870336	DAG1	AG	49557857
rs3886934	MMP2	AG	55446764
rs3892767	ADAM12	CT	128041169
rs3911239	ITGAV	CT	187470311
rs3918261	MMP9	AG	44643592
rs3918278	MMP9	AG	44635654
rs3920038	NID2	GT	52535648
rs3922914	COL5A1	CT	137598731
rs3924229	BMP1	AG	22049892
rs3924462	DAG1	GT	49524236
rs3925075	ITGAM	AG	31347748
rs3929758	COL4A2	AC	111082157
rs3930345	VCAN	CT	82881255
rs3933239	MMP9	AC	44628668
rs3935335	COL5A1	AG	137596166
rs3943300	LAMA2	CT	129454510
rs3995780	ADAM12	CT	128011731

rs4043263	ADAM11	AG	42860326
rs4045192	ADAM15	CT	155038756
rs4072037	THBS3	CT	155162067
rs4072077	COL5A1	GT	137677489
rs4073904	COL4A3	AG	228161915
rs4075478	BMP1	CT	22053123
rs4076090	LAMA2	AG	129351484
rs4077920	MATN2	AC	98893864
rs4129319	SPARCL1	CT	88395385
rs4141663	ITGB5	CT	124551967
rs4143750	LAMA2	AG	129734028
rs4145072	COL4A1	CT	110899955
rs4233366	ADAMTS4	CT	161159147
rs4233367	ADAMTS4	CT	161163037
rs4233964	EFEMP1	CT	56135497
rs4234222	ITGB5	AG	124584787
rs4240702	COL5A1	CT	137595859
rs4240703	COL5A1	AG	137595928
rs4242430	BMP1	AC	22067769
rs4246818	COL23A1	AC	177798187
rs4255644	COL4A2	AG	111089573
rs4255736	FBN1	AC	48728291
rs4260765	LAMA2	CT	129217305
rs4263106	COL4A3	GT	228092899
rs4271760	COL4A3	AG	228167544
rs4279134	LAMB2	AG	49156676
rs4279641	COL14A1	AG	121283832
rs4280978	LAMA2	GT	129619236
rs4285344	LAMA2	AG	129219050
rs4290780	CCDC80	AG	112335039
rs4292545	LAMA2	AC	129269412
rs4325745	COL4A3	CT	228057792
rs4332918	COL4A3	AG	228124702
rs4335205	COL5A1	AG	137552398
rs4338381	COL11A1	AG	103572927
rs4340680	CCDC80	AC	112332629
rs4340795	NPNT	AG	106861730
rs4341231	COL5A1	CT	137592961
rs4342076	PLOD2	GT	145888493
rs4347830	COL4A3	AG	228058016
rs4349734	COL23A1	CT	177845760
rs4357054	COL23A1	AG	177860454
rs4360265	MATN2	CT	98889153
rs4361533	COL23A1	CT	177851623
rs4367177	NPNT	CT	106921594
rs4370848	ADAM12	AC	128085524
rs4390761	COL4A3	CT	228156969
rs4392253	COL4A3	AC	228079100

rs4394301	FKBP9	AG	33049584
rs4405983	SPARCL1	CT	88386769
rs4407920	COL14A1	AG	121141973
rs4426386	ADAM11	AG	42835495
rs4428739	COL5A1	CT	137736729
rs4430946	COL4A3	CT	228078708
rs4431992	MMP10	CT	102645399
rs4433949	COL6A3	CT	238249630
rs4443524	LAMA2	AC	129205288
rs4447275	COL4A2	AG	110982419
rs4461027	LOXL1	CT	74211548
rs4461616	ADAM19	GT	156981913
rs4470745	VCAN	AG	82789647
rs4485771	TLL1	AG	166893533
rs4504708	COL5A1	GT	137736544
rs4512966	COL4A2	CT	111082058
rs4517640	COL4A2	CT	111075916
rs4528750	COL4A3	AC	228058048
rs4531123	COL5A1	AG	137701553
rs4534007	LAMA2	AG	129373689
rs4534508	TLL2	CT	98282986
rs4548258	COL5A1	CT	137599444
rs4559216	MATN2	AG	98894110
rs4563961	COL5A1	CT	137670002
rs4565825	ADAM12	AG	127984277
rs4569989	LAMA2	AG	129408902
rs4571602	LAMA4	AG	112576955
rs4575098	ADAMTS4	AG	161155392
rs4587680	ITGB1	CT	33200782
rs4588897	COL14A1	AG	121139142
rs4596126	FBLN2	AC	13659897
rs4596720	COL5A1	AG	137587628
rs4597836	NPNT	AC	106888263
rs4600917	NPNT	CT	106829645
rs4607021	ITGB2	AG	46322487
rs4610302	SPARCL1	AG	88400110
rs4616261	CSPG4	CT	75965169
rs4619602	COL4A3	CT	228167014
rs4631429	MATN2	AG	98876595
rs4642505	LAMA2	AG	129642470
rs4651138	LAMC1	AC	183001312
rs4651322	TNN	CT	175104719
rs4652769	LAMC1	CT	183009196
rs4656614	DPT	AG	168691293
rs4658260	TGFBR3	AG	92184673
rs4658265	TGFBR3	CT	92240685
rs4658269	TGFBR3	GT	92247654
rs4659618	NID1	AG	236150459

rs4659620	NID1	GT	236201609
rs4660139	NID1	CT	236155817
rs4660141	NID1	AG	236162592
rs4660151	NID1	AG	236212899
rs4663257	COL6A3	AG	238313196
rs4663724	COL6A3	AC	238235082
rs4663731	COL6A3	AG	238267058
rs4667257	COL3A1	AG	189880810
rs4669632	PDIA6	CT	10971017
rs4669633	PDIA6	AG	10976790
rs4675157	COL4A3	GT	228103702
rs4675159	COL4A3	AG	228109347
rs4675161	COL4A3	AG	228127807
rs4678164	ITGB5	AG	124525071
rs4678167	ITGB5	CT	124540832
rs4678169	ITGB5	AC	124543103
rs4681298	PLOD2	CT	145844373
rs4682417	CCDC80	AG	112350156
rs4684147	FBLN2	CT	13621532
rs4690833	TLL1	GT	167016664
rs4693829	SPARCL1	AC	88438011
rs4693833	SPARCL1	GT	88445658
rs4704742	ADAM19	CT	156891353
rs4704863	ADAM19	CT	156879727
rs4704869	ADAM19	AG	156898899
rs4717865	ELN	AG	73454199
rs4720087	FKBP9	AG	32995331
rs4727007	PDIA4	AG	148709810
rs4727695	LAMB1	AG	107614003
rs4728730	ADAM22	CT	87704972
rs4729129	COL1A2	AG	94015393
rs4735508	MATN2	AG	98917811
rs4735518	MATN2	GT	98991719
rs4735521	MATN2	CT	99008015
rs4743307	COL15A1	AG	101764212
rs4743322	COL15A1	AC	101835165
rs4756776	SPON1	AGT	14224883
rs4756782	SPON1	AC	14254606
rs4760607	COL2A1	GT	48365265
rs4760674	COL2A1	AC	48357014
rs4771662	COL4A1	AG	110946729
rs4771685	COL4A2	AG	111168951
rs4773139	COL4A1	AG	110932193
rs4773155	COL4A2	AC	110971066
rs4773179	COL4A2	AG	111061884
rs4773184	COL4A2	CT	111080231
rs4773191	COL4A2	CT	111126501
rs4773194	COL4A2	AG	111132490

rs4773200	COL4A2	CT	111156021
rs4773201	COL4A2	AG	111156933
rs4774517	FBN1	GT	48759291
rs4783891	MMP2	AG	55457356
rs4789863	TIMP2	CT	76897347
rs4789906	LGALS3BP	AG	76974752
rs4789907	LGALS3BP	AG	76964309
rs4789908	LGALS3BP	CT	76962059
rs4789910	LGALS3BP	GT	76959898
rs4789933	TIMP2	CT	76923426
rs4789936	TIMP2	CT	76897974
rs4789939	TIMP2	CT	76881703
rs4789940	TIMP2	AC	76877671
rs4796812	TIMP2	CT	76875186
rs4798032	EMILIN2	CT	2854002
rs4810482	MMP9	CT	44634550
rs4818817	COL6A1	AG	47433055
rs4818989	ITGB2	AG	46356816
rs4819095	COL18A1	CT	46817380
rs4819099	COL18A1	AG	46834814
rs4819119	COL18A1	AG	46883153
rs4819179	COL6A1	CT	47419012
rs4820294	LGALS1	AG	38071043
rs4841924	COL5A1	AG	137587768
rs4841926	COL5A1	CT	137601285
rs4841931	COL5A1	CT	137705289
rs4841934	COL5A1	CT	137707942
rs4842139	COL5A1	CT	137563692
rs4842145	COL5A1	CT	137596949
rs4842151	COL5A1	CT	137646511
rs4842152	COL5A1	AG	137647009
rs4842153	COL5A1	CT	137647314
rs4842157	COL5A1	AG	137658608
rs4842158	COL5A1	AG	137658722
rs4842161	COL5A1	AC	137659688
rs4842163	COL5A1	CT	137662741
rs4842167	COL5A1	CT	137687314
rs4842168	COL5A1	CT	137691346
rs4842169	COL5A1	AG	137699002
rs4842173	COL5A1	CT	137718113
rs4842174	COL5A1	CT	137722311
rs4845710	ADAM15	AG	155033014
rs4870723	COL14A1	AC	121228679
rs4870728	COL14A1	CT	121386925
rs4871046	COL14A1	CT	121177395
rs4871829	COL14A1	AG	121081289
rs4871830	COL14A1	AG	121081936
rs4872360	BMP1	CT	22028668

rs4886727	CSPG4	CT	75957375
rs4886728	CSPG4	AG	75961518
rs4886776	LOXL1	AG	74224996
rs4886782	LOXL1	AG	74228810
rs4889640	ITGAM	AC	31264267
rs4895840	LAMA2	CT	129344023
rs4895841	LAMA2	CT	129344194
rs4895846	LAMA2	CT	129384367
rs4897323	LAMA2	AC	129756916
rs4897554	CTGF	AG	132262991
rs4904827	FBLN5	CT	92401585
rs4905211	SERPINA12	AG	94961888
rs4919029	TLL2	AG	98274045
rs4932432	ACAN	AC	89372601
rs4932435	ACAN	GT	89384393
rs4932438	ACAN	ACGT	89397057
rs4932439	ACAN	AG	89401109
rs4947172	LAMA4	CT	112501537
rs4947179	LAMA4	AC	112549164
rs4958484	SPARC	AG	151052146
rs4958486	SPARC	AC	151058334
rs4958487	SPARC	AG	151063674
rs4962318	ADAM12	AG	127741144
rs4962322	ADAM12	AC	127932765
rs4962333	ADAM12	CT	127971952
rs4962510	ADAM12	AG	127850143
rs4962514	ADAM12	AG	127881422
rs4962553	ADAM12	GT	128086622
rs4971100	THBS3	AG	155155731
rs4974641	SPON2	AG	1174861
rs4976725	COL23A1	CT	177876309
rs4976733	COL23A1	CT	177968251
rs4976761	COL23A1	AC	177766183
rs4976765	COL23A1	AG	177789202
rs4976783	COL23A1	AG	177901202
rs4976788	COL23A1	CT	177960432
rs4976790	COL23A1	GT	177968915
rs4982695	MMP14	CT	23322613
rs4982697	MMP14	AG	23326242
rs4999923	TIMP3	CT	33251408
rs5012370	MATN2	CT	98938231
rs5024595	ADAM12	CT	127856798
rs5025055	TGFBR3	CT	92141816
rs5749524	TIMP3	CT	33224285
rs5754289	TIMP3	CT	33192544
rs5764781	FBLN1	AG	45943677
rs5764804	FBLN1	CT	45990039
rs5765425	FBLN1	AG	45892433

rs5765475	FBLN1	CT	45959337
rs5765512	FBLN1	CT	45983415
rs5929098	COL4A5	CT	107692305
rs5929100	COL4A5	AG	107711906
rs5929126	COL4A5	AC	107690996
rs5973878	COL4A5	GT	107715484
rs5987017	BGN	AG	152782172
rs6007085	FBLN1	GT	45936527
rs6007088	FBLN1	AG	45949291
rs6104416	MMP9	AC	44628514
rs6130803	MATN4	AG	43923102
rs6422346	COL23A1	AG	177678526
rs6422347	COL23A1	CT	177863083
rs6424883	LAMC1	CT	183037695
rs6427128	ADAM15	AC	155026942
rs6429452	NID1	CT	236155219
rs6429466	NID1	CT	236190813
rs6432017	ADAM17	AG	9693875
rs6434304	COL3A1	CT	189833911
rs6434309	COL3A1	AC	189883166
rs6434317	COL5A2	GT	189936687
rs6436661	COL4A3	CT	228056670
rs6436664	COL4A3	CT	228084164
rs6436666	COL4A3	AG	228092697
rs6436669	COL4A3	AG	228113175
rs6436670	COL4A3	CT	228126494
rs6437343	GPC1	AG	241367465
rs6438087	CCDC80	AG	112366816
rs6442396	FBLN2	AG	13586461
rs6453815	COL12A1	AG	75835305
rs6464929	PDIA4	AG	148717467
rs6464930	PDIA4	CT	148720906
rs6465412	COL1A2	CT	94058395
rs6468601	MATN2	CT	98877537
rs6469915	COL14A1	AG	121325670
rs6469916	COL14A1	AG	121332791
rs6478963	COL15A1	CT	101708150
rs6478966	COL15A1	AG	101776125
rs6480654	P4HA1	AC	74773230
rs6481819	ITGB1	CT	33299459
rs6486166	SPON1	CT	14105497
rs6486169	SPON1	CT	14155002
rs6486178	SPON1	CT	14206679
rs6486182	SPON1	AG	14233522
rs6492246	COL4A1	CT	110869422
rs6492252	COL4A1	AG	110922694
rs6492270	COL4A2	AG	111072732
rs6492279	COL4A2	CT	111165390

rs6493327	FBN1	AG	48809428
rs6501266	TIMP2	CT	76907353
rs6520278	TIMP1	CT	47447924
rs6520279	TIMP1	CT	47448096
rs6536939	TLL1	AG	166889110
rs6536942	TLL1	AG	166986194
rs6537935	COL5A1	CT	137524648
rs6537941	COL5A1	AC	137559417
rs6537949	COL5A1	GT	137673861
rs6563562	POSTN	CT	38158718
rs6569585	LAMA2	CT	129474946
rs6569603	LAMA2	AC	129793717
rs6572807	NID2	AG	52480621
rs6572813	NID2	CT	52539090
rs6575439	SERPINA12	CT	94980807
rs6579891	SPARC	CT	151074609
rs6580647	COL2A1	AC	48403229
rs6584079	TLL2	CT	98252496
rs6584080	TLL2	CT	98255951
rs6597740	ADAM12	CT	127852721
rs6597743	ADAM12	CT	127879856
rs6597745	ADAM12	CT	127895098
rs6597750	ADAM12	AC	127943038
rs6597753	ADAM12	CT	127948079
rs6597754	ADAM12	CT	127948238
rs6605524	THBS2	AG	169656120
rs6651180	MATN2	CT	99018395
rs6660484	TGFBR3	AG	92172608
rs6665312	TGFBR3	CT	92180903
rs6667609	COL11A1	GT	103499049
rs6667611	TNN	CT	175033141
rs6677523	TGFBR3	AG	92202467
rs6680614	TGFBR3	GT	92358371
rs6682554	PLOD1	CT	11986621
rs6683840	TGFBR3	AG	92249817
rs6690340	LAMC1	AG	183116686
rs6691075	COL11A1	AG	103416690
rs6693438	TGFBR3	AG	92163174
rs6696455	TNN	CT	175087729
rs6697630	FMOD	AG	203319816
rs6697996	PLOD1	GT	12006802
rs6701037	TNN	AC	175120079
rs6703740	TNN	GT	175041977
rs6704864	COL4A3	AG	228051314
rs6705408	ADAM17	CT	9630231
rs6707530	FN1	GT	216240584
rs6718820	COL4A3	AC	228038804
rs6722825	COL4A3	AG	228039866

rs6723547	COL4A3	CT	228098122
rs6726181	EFEMP1	CT	56087766
rs6727477	COL4A3	CT	228059671
rs6728149	COL4A3	AG	228092334
rs6728818	COL6A3	AG	238244963
rs6729152	COL4A3	GT	228144706
rs6735744	COL4A3	CT	228036202
rs6737679	COL4A3	AG	228059546
rs6737889	COL4A3	AG	228052220
rs6739977	COL4A3	AG	228065095
rs6741369	ITGAV	GT	187487269
rs6744212	COL6A3	AG	238224335
rs6744921	FN1	AG	216266736
rs6756117	COL4A3	AG	228069010
rs6756834	COL4A3	CT	228083236
rs6756898	COL4A3	CT	228092406
rs6758526	COL6A3	AG	238327751
rs6759927	COL6A3	AG	238227919
rs6765409	FBLN2	CT	13646247
rs6768588	ITGB5	AG	124487335
rs6771896	ITGB5	AG	124545750
rs6774776	CCDC80	AG	112345135
rs6779394	LAMB2	AG	49157771
rs6787638	ITGB5	CT	124582319
rs6794305	CCDC80	CT	112355977
rs6801418	PLOD2	AC	145833232
rs6801891	FBLN2	CT	13667818
rs6803572	FBLN2	AG	13650434
rs6807798	CCDC80	GT	112359926
rs6811456	TLL1	CT	166785679
rs6817700	NPNT	AG	106891531
rs6839829	SPARCL1	CT	88454048
rs6843517	NPNT	AG	106870409
rs6855132	NPNT	AG	106877631
rs6860507	ADAM19	AG	156898690
rs6861486	SPARC	CT	151049775
rs6861887	SPARC	GT	151050044
rs6861956	TGFBI	CT	135409014
rs6867396	VCAN	AG	82776301
rs6875485	ADAM19	AC	156966595
rs6877684	SPARC	AC	151033821
rs6880837	TGFBI	CT	135396669
rs6892747	COL23A1	CT	177715452
rs6894906	TGFBI	AG	135355038
rs6899012	TGFBI	AG	135403529
rs6899205	ADAM19	AG	156943285
rs6899453	LAMA4	GT	112500496
rs6907109	LAMA4	CT	112502989

rs6912145	LAMA4	AG	112519743
rs6913656	LAMA4	AG	112483235
rs6914003	LAMA2	CT	129715822
rs6917532	LAMA2	AG	129788658
rs6917644	CTGF	AG	132265665
rs6921064	LAMA2	AC	129410662
rs6926573	LAMA4	CT	112564517
rs6930262	LAMA4	AG	112427825
rs6934624	LAMA2	AG	129413333
rs6936981	LAMA2	CT	129667270
rs6939307	LAMA2	GT	129838002
rs6940420	THBS2	AG	169631907
rs6940801	THBS2	AG	169655667
rs6943225	LAMB1	CT	107646766
rs6950982	SERPINE1	AG	100766603
rs6958024	ADAM22	GT	87695787
rs6968026	ADAM22	AG	87713925
rs6979091	LAMB1	GT	107651361
rs6988293	COL14A1	AG	121234298
rs6989074	COL14A1	AG	121234756
rs6996243	MATN2	AG	98871690
rs7001798	COL14A1	AC	121336915
rs7004099	COL14A1	AC	121325031
rs7005932	COL14A1	AG	121194900
rs7013298	COL14A1	CT	121087046
rs7018223	COL14A1	AG	121214976
rs7031588	COL15A1	CT	101822302
rs7035322	TNC	AC	117787173
rs7044529	COL5A1	CT	137568051
rs7053892	COL4A5	AG	107675049
rs7062216	BGN	AG	152769502
rs7069895	ADAM12	AG	127913896
rs7070439	TLL2	AG	98230489
rs7070979	TLL2	CT	98170594
rs7072808	ADAM12	CT	127964149
rs7078184	TLL2	AG	98250191
rs7078304	TLL2	AG	98183070
rs7078493	TLL2	GT	98204095
rs7079180	ADAM12	CT	127790305
rs7080604	ADAM12	AG	127764957
rs7081193	ADAM12	CT	128074225
rs7082031	ADAM12	CT	128038717
rs7083245	TLL2	CT	98221417
rs7084288	ADAM12	AG	128059654
rs7089454	ADAM12	CT	127904710
rs7097346	ADAM12	AG	128007683
rs7097356	ADAM12	CT	127911584
rs7099756	TLL2	CT	98252519

rs7099772	ADAM12	CT	127729222
rs7103919	SPON1	CT	14253351
rs7104613	SPON1	CT	14079931
rs7109409	SPON1	AG	14167577
rs7119081	SPON1	AG	14220811
rs7119084	MMP10	AG	102649235
rs7124311	SPON1	GT	14050389
rs7125320	MMP1	GT	102663708
rs7139699	COL4A2	CT	111005475
rs7140030	COL4A2	AG	111064159
rs7143288	FBLN5	CT	92337877
rs7144352	NID2	CT	52495375
rs7152296	SERPINA12	AG	94960384
rs7158068	SERPINA12	CT	94973644
rs7161648	SERPINA12	CT	94965464
rs7164585	FBN1	CT	48827144
rs7165060	FBN1	CT	48787194
rs7169625	FBN1	CT	48739652
rs7169848	FBN1	CT	48879360
rs7173049	LOXL1	AG	74244610
rs7180356	ACAN	CT	89371887
rs7183203	FBN1	CT	48870456
rs7187242	MMP2	CT	55499994
rs7187536	MMP2	AG	55491927
rs7190378	MMP2	AG	55469404
rs7193268	ITGAM	CT	31340997
rs7197377	MMP2	AG	55440604
rs7199709	MMP2	CT	55436813
rs7212662	TIMP2	GT	76918131
rs7214592	TIMP2	AG	76930769
rs7218237	TIMP2	GT	76871638
rs7222639	LGALS3BP	CT	76958759
rs7226712	EMILIN2	CT	2874552
rs7250381	CALR	AG	13050201
rs7278425	COL18A1	CT	46926551
rs7280485	COL6A2	AG	47548347
rs7283829	COL6A1	AG	47427839
rs7284208	FBLN1	AG	45950803
rs7287772	TIMP3	CT	33188307
rs7305954	COL2A1	CT	48403530
rs7317390	COL4A2	CT	110984977
rs7317733	COL4A2	AC	111146513
rs7317784	COL4A1	CT	110955074
rs7317784	COL4A2	CT	110955074
rs7318486	COL4A2	AC	111013203
rs7319307	COL4A1	CT	110793077
rs7319311	COL4A2	AG	111030578
rs7322495	COL4A2	CT	110991285

rs7323378	POSTN	CT	38153351
rs7326145	COL4A2	AC	111047350
rs7326449	COL4A2	AG	111099122
rs7327721	COL4A2	CT	111174526
rs7328731	COL4A2	AG	111056994
rs7330054	COL4A2	AG	110986632
rs7330851	COL4A1	AG	110844188
rs7334322	COL4A2	CT	111150714
rs7336547	COL4A2	CT	110992985
rs7342880	TIMP2	AC	76874512
rs7358800	COL4A2	AG	111012857
rs7373878	ITGB5	GT	124610238
rs7380466	COL23A1	AG	177720154
rs7419543	COL5A2	CT	189967433
rs7422977	COL3A1	GT	189884587
rs7445039	COL23A1	AG	177795627
rs7453910	LAMA2	CT	129678863
rs7483963	SPON1	AG	14235676
rs7501702	MFAP4	AG	19293727
rs7502935	TIMP2	AG	76878629
rs7513205	ECM1	AG	150472781
rs7514724	TGFBR3	CT	92173847
rs7517044	TGFBR3	AG	92198162
rs7536947	TGFBR3	CT	92248695
rs7537288	COL11A1	GT	103576354
rs7541061	CHI3L1	AC	203163367
rs7542028	DPT	CT	168687822
rs7542294	CHI3L1	AG	203151176
rs7543148	FMOD	CT	203317162
rs7543626	COL11A1	AG	103578334
rs7544130	COL11A1	AC	103451455
rs7544816	COL11A1	AC	103556059
rs7546260	TNN	GT	175086965
rs7550034	TGFBR3	AG	92364576
rs7555476	TGFBR3	CT	92310666
rs7555548	TGFBR3	GT	92310690
rs7559906	EFEMP1	CT	56133040
rs7561588	ADAM17	AG	9619204
rs7563085	EFEMP1	AG	56103170
rs7575234	FN1	CT	216268902
rs7576849	GPC1	AG	241386352
rs7577243	GPC1	AG	241382083
rs7577732	COL4A3	AG	228098933
rs7579903	COL3A1	AG	189862097
rs7586225	COL6A3	AC	238255686
rs7587228	COL4A3	CT	228177479
rs7588661	FN1	CT	216253454
rs7589466	EFEMP1	AG	56103352

rs7596514	COL3A1	AG	189878769
rs7597795	COL6A3	AG	238270726
rs7604984	FN1	AG	216217639
rs7606754	COL4A3	AG	228135180
rs7606877	GPC1	AG	241410024
rs7607614	COL3A1	CT	189841052
rs7610284	FBLN2	CT	13680861
rs7620601	CCDC80	CT	112371578
rs7650893	ITGB5	CT	124533315
rs7654070	TLL1	AG	166854660
rs7659624	SPON2	CT	1179213
rs7669159	TLL1	AG	166939490
rs7669287	SPON2	AG	1159680
rs7677557	NPNT	CT	106872861
rs7679471	TLL1	CT	166854959
rs7683311	SPARCL1	AC	88392755
rs7691244	NPNT	AG	106901228
rs7704172	COL23A1	CT	177727491
rs7706759	COL23A1	AG	177893248
rs7708757	COL23A1	CT	177777888
rs7710491	VCAN	CT	82778462
rs7713018	COL23A1	GT	177956908
rs7713983	COL23A1	CT	177690978
rs7714353	ADAM19	CT	156870534
rs7715762	COL23A1	AG	177664297
rs7716953	COL23A1	CT	177842788
rs7718220	COL23A1	CT	177919951
rs7719224	ADAM19	CT	156999340
rs7719352	COL23A1	CT	177661902
rs7719521	SPARC	AC	151049404
rs7722612	COL23A1	CT	177826469
rs7724199	ADAM19	AG	156989957
rs7725069	ADAM19	CT	156989757
rs7727323	COL23A1	AG	177780183
rs7728076	COL23A1	CT	177857534
rs7729589	COL23A1	CT	177787515
rs7738951	LAMA4	AG	112518090
rs7741996	LAMA2	CT	129796620
rs7742931	LAMA4	ACG	112463693
rs7751685	LAMA2	AC	129422405
rs7753862	LAMA2	CT	129646393
rs7754167	LAMA2	AG	129823645
rs7754329	LAMA4	CT	112526404
rs7758152	COL12A1	CT	75815840
rs7763896	CTGF	GT	132279804
rs7764005	THBS2	CT	169607718
rs7766236	LAMA4	AC	112485798
rs7766548	LAMA2	AG	129525473

rs7766689	LAMA2	AG	129643527
rs7766787	LAMA4	AG	112573780
rs7770270	LAMA2	AG	129707118
rs7774197	TNXB	AC	32046275
rs7777930	PLOD3	CT	100853231
rs7788778	LAMB1	CT	107601523
rs7789846	LAMB1	CT	107605412
rs7792913	ADAM22	CT	87812968
rs7805430	COL1A2	AG	94056216
rs7810426	ADAM22	GT	87826957
rs7812993	BMP1	AG	22025644
rs7813088	COL14A1	AC	121269836
rs7813506	COL14A1	AG	121270124
rs7814222	COL14A1	AG	121270355
rs7814885	BMP1	CT	22063131
rs7815790	MATN2	AG	98902730
rs7819160	COL14A1	AG	121329119
rs7819541	BMP1	GT	22042151
rs7820004	COL14A1	CT	121140963
rs7827219	COL14A1	AG	121130164
rs7831897	MATN2	AG	98900769
rs7838961	BMP1	AG	22038952
rs7841621	COL14A1	CT	121068248
rs7843130	COL14A1	AG	121120890
rs7849193	COL5A1	AG	137650824
rs7849777	COL5A1	AG	137672801
rs7851787	COL15A1	CT	101785633
rs7855580	COL5A1	AG	137661911
rs7863250	COL15A1	CT	101793834
rs7864699	COL5A1	CT	137724371
rs7869742	ECM2	AG	95256174
rs7875140	COL5A1	CT	137613772
rs7896870	ADAM12	CT	127827684
rs7897850	ADAM12	GT	127693203
rs7900158	ADAM12	CT	127718332
rs7900442	ITGB1	AG	33186544
rs7901239	ADAM12	CT	128073227
rs7901456	ADAM12	AC	127970386
rs7908946	ADAM12	AG	127913568
rs7910333	ADAM12	CT	128030262
rs7911793	ADAM12	CT	127731535
rs7912236	ADAM12	CT	127879242
rs7915341	TLL2	GT	98157460
rs7919721	TLL2	AG	98158494
rs7920091	ADAM12	AG	127722422
rs7932425	SPON1	CT	14242470
rs7945189	MMP1	CT	102660564
rs7945189	MMP10	CT	102660564

rs7948160	SPON1	AG	14261886
rs7954702	DCN	CT	91530866
rs7971880	COL2A1	AG	48391055
rs7981946	COL4A1	CT	110812242
rs7987982	COL4A1	CT	110931311
rs7989565	COL4A2	AG	110982357
rs7991229	COL4A2	GT	111091995
rs7991436	COL4A2	AG	111026440
rs7994305	COL4A1	AC	110868460
rs7995158	COL4A2	AG	111111717
rs7995370	COL4A2	CT	111118948
rs7999790	COL4A1	GT	110895995
rs8004587	SERPINA12	AG	94987996
rs8008729	SERPINA12	CT	94950121
rs8014455	NID2	CT	52461699
rs8014548	FBLN5	CT	92409103
rs8024016	ACAN	GT	89408081
rs8028537	ACAN	AG	89345947
rs8031532	ACAN	GT	89358329
rs8031741	ACAN	AG	89418869
rs8036376	THBS1	AC	39868627
rs8040336	PDIA3	CT	44053617
rs8041685	LOXL1	AG	74225193
rs8048583	ITGAM	CT	31279536
rs8054459	MMP2	AG	55544549
rs8064954	ADAM11	AC	42867443
rs8066116	TIMP2	CT	76929879
rs8074370	LGALS3BP	CT	76957331
rs8080307	TIMP2	AG	76858353
rs8097282	EMILIN2	AG	2878613
rs8123253	MATN4	CT	43912390
rs8126757	COL18A1	AG	46848282
rs8130155	ADAMTS1	CT	28226646
rs8136803	TIMP3	GT	33237112
rs8138025	FBLN1	AG	45974757
rs8179334	COL11A1	AG	103367095
rs8192306	BMP1	AG	22015853
rs9283850	THBS2	AG	169637456
rs9285464	LAMA2	CT	129314221
rs9285465	LAMA2	CT	129734216
rs9288163	COL5A2	AG	190013146
rs9289713	PLOD2	CT	145824025
rs9293996	COL12A1	CT	75826134
rs9294977	THBS2	AG	169624172
rs9301432	COL4A1	AG	110811451
rs9301436	COL4A1	AG	110873099
rs9301445	COL4A1	CT	110951487
rs9301445	COL4A2	CT	110951487

rs9302667	MMP2	AC	55456835
rs9302671	MMP2	GT	55521725
rs9305294	ADAMTS1	CT	28225582
rs9306126	COL18A1	CT	46816363
rs9313634	ADAM19	CT	156964594
rs9315503	POSTN	AG	38139260
rs9321159	LAMA2	CT	129630354
rs9374311	LAMA4	CT	112524266
rs9375609	LAMA2	CT	129346877
rs9379341	THBS2	CT	169643685
rs9388686	LAMA2	AC	129425564
rs9388700	LAMA2	CT	129697797
rs9398302	LAMA4	AG	112556666
rs9398901	LAMA2	AG	129671311
rs9399005	CTGF	CT	132268964
rs9402089	LAMA2	CT	129275826
rs9402090	LAMA2	CT	129313499
rs9402098	LAMA2	AG	129371533
rs9402130	LAMA2	AG	129748503
rs9410002	COL5A1	AG	137730956
rs9422812	ADAM12	GT	127834139
rs9422969	ADAM12	AG	127997303
rs9447453	COL12A1	AC	75878425
rs9482954	LAMA2	GT	129235633
rs9482965	LAMA2	AG	129283603
rs9482982	LAMA2	GT	129405133
rs9483012	LAMA2	AC	129678523
rs9483364	CTGF	AG	132281798
rs9487847	LAMA4	CT	112529268
rs9487853	LAMA4	CT	112538321
rs9487861	LAMA4	AC	112558498
rs9492168	LAMA2	CT	129295840
rs9492188	LAMA2	CT	129332591
rs9492191	LAMA2	AG	129335259
rs9492200	LAMA2	AG	129344515
rs9492223	LAMA2	AG	129404742
rs9492262	LAMA2	CT	129505832
rs9492268	LAMA2	ACT	129513448
rs9492325	LAMA2	AG	129788465
rs9505896	THBS2	AG	169646920
rs9505933	THBS2	CT	169606443
rs9515168	COL4A1	GT	110869433
rs9515170	COL4A1	CT	110878775
rs9515201	COL4A2	AC	111040798
rs9521623	COL4A1	AC	110809382
rs9521642	COL4A1	AG	110845060
rs9521650	COL4A1	AG	110866265
rs9521664	COL4A1	CT	110889902

rs9521666	COL4A1	AG	110897444
rs9521699	COL4A2	AG	110984609
rs9521725	COL4A2	AG	111024974
rs9521733	COL4A2	CT	111034542
rs9521748	COL4A2	AG	111062680
rs9521783	COL4A2	AG	111111670
rs9521789	COL4A2	CT	111119620
rs9521803	COL4A2	CT	111132556
rs9547947	POSTN	AC	38131993
rs9547952	POSTN	CT	38138689
rs9555680	COL4A1	CT	110921814
rs9555682	COL4A1	AG	110941977
rs9555690	COL4A2	CT	111013190
rs9555701	COL4A2	GT	111103231
rs9555714	COL4A2	AG	111151959
rs9559771	COL4A2	AG	110985222
rs9559792	COL4A2	CT	111059093
rs9559823	COL4A2	AG	111143101
rs9583467	COL4A1	AG	110860861
rs9583484	COL4A1	GT	110966349
rs9583484	COL4A2	GT	110966349
rs9588148	COL4A2	GT	111021081
rs9603226	POSTN	AG	38143586
rs9609643	TIMP3	AG	33251059
rs9619311	TIMP3	CT	33196693
rs9630453	ACAN	AG	89403922
rs9636786	ADAMTS1	CT	28209494
rs9637198	COL18A1	CT	46820082
rs9637646	TLL1	CT	166803528
rs9641021	ADAM22	CT	87666666
rs9641023	ADAM22	GT	87759090
rs9646148	MMP14	GT	23300095
rs9659030	COL11A1	CT	103342392
rs9661103	TGFBR3	CT	92335906
rs9704394	SPON1	AG	13997399
rs9717830	THBS2	CT	169663638
rs9765848	THBS2	CT	169649827
rs9792164	MATN2	AG	98919139
rs9806163	FBN1	CT	48803839
rs9820945	PLOD2	CT	145818406
rs9821338	CCDC80	CT	112375450
rs9822704	FBLN2	CT	13603144
rs9831797	FBLN2	CT	13601079
rs9835273	FBLN2	AG	13653400
rs9838270	FBLN2	CT	13676940
rs9843344	FBLN2	AG	13670536
rs9855853	CCDC80	AG	112339751
rs9863413	FBLN2	AG	13651835

rs9870432	CCDC80	AC	112341941
rs9874118	ITGB5	CT	124516793
rs9879289	ITGB5	AG	124498173
rs9880088	LAMB2	AG	49178990
rs9881100	FBLN2	AG	13582768
rs9888739	ITGAM	CT	31313253
rs9889410	TIMP2	AG	76878980
rs9894295	TIMP2	AG	76862105
rs9900972	TIMP2	AG	76868614
rs9917580	FBLN1	AG	45907687
rs9921970	MMP2	AG	55445343
rs9933802	MMP2	GT	55429134
rs9937837	ITGAM	GT	31298939
rs9950998	EMILIN2	CT	2872368
rs9968182	ITGB5	CT	124601972
rs9969584	COL14A1	AG	121104786
rs9970726	NID1	AC	236132627
rs9976834	COL18A1	CT	46897214
rs9977482	COL18A1	CT	46899119
rs9978018	COL6A2	AG	47526240
rs9980531	COL18A1	AG	46859717
rs9981981	COL6A2	AG	47544838
rs9982817	COL6A2	CT	47514885
rs9990589	TLL1	AG	166830580
rs9992768	TLL1	AG	166901466
rs9993980	SPARCL1	CT	88424737
rs10000995	NPNT	CT	106839018
rs10001608	SPARCL1	CT	88437710
rs10001634	NPNT	AG	106895964
rs10003271	TLL1	GT	166945293
rs10003497	SPARCL1	AG	88429759
rs10024099	SPARCL1	AG	88389081
rs10027979	SPARCL1	CT	88456441
rs10037451	COL23A1	CT	177856605
rs10039491	COL23A1	CT	177891489
rs10040971	LOX	CT	121404787
rs10046552	COL1A2	AC	94057418
rs10065788	ADAM19	CT	156849535
rs10067096	ADAM19	AC	156917340
rs10070303	COL23A1	CT	177840382
rs10087151	COL14A1	AG	121172261
rs10098159	MATN2	AG	99009191
rs10099863	MATN2	AC	98874693
rs10104973	COL14A1	AG	121104385
rs10110722	COL14A1	AG	121178112
rs10111291	COL14A1	AG	121266654
rs10117421	TNC	AC	117778369
rs10118040	TNC	AG	117879414

rs10120210	ECM2	GT	95284982
rs10125663	TNC	CT	117886416
rs10128229	ADAM12	AG	127982362
rs10137158	NID2	CT	52509858
rs10140797	SERPINA12	AC	94965685
rs10142528	FBLN5	CT	92330790
rs10145558	SERPINA12	AG	94971978
rs10146057	NID2	AG	52480752
rs10146335	FBLN5	AG	92423309
rs10149012	FBLN5	CT	92332816
rs10153222	MMP2	GT	55436751
rs10161783	COL4A2	CT	111009055
rs10164812	COL4A3	CT	228090011
rs10167850	COL6A3	AC	238271284
rs10178458	COL4A3	CT	228111435
rs10178599	COL6A3	AG	238264223
rs10179642	ADAM17	CT	9683696
rs10187805	COL4A3	AG	228100150
rs10194682	COL5A2	AG	189906743
rs10197595	PDIA6	AG	10956867
rs10197695	PDIA6	AG	10926910
rs10198349	COL4A3	CT	228061398
rs10200215	COL5A2	CT	189904674
rs10202434	COL6A3	GT	238251035
rs10202483	FN1	AG	216297875
rs10207952	COL4A3	AG	228063979
rs10220027	COL4A2	CT	111173659
rs10229305	LAMB1	CT	107638236
rs10229877	ADAM22	AG	87750115
rs10235102	COL1A2	AC	94051795
rs10238083	ADAM22	AG	87721928
rs10239100	ADAM22	AC	87564277
rs10268574	ADAM22	GT	87768174
rs10279744	ADAM22	AG	87670741
rs10282572	ADAM22	CT	87674671
rs10399805	CHI3L1	AG	203155998
rs10406152	COL5A3	CT	10071063
rs10412065	COL5A3	CT	10093028
rs10447440	LAMA2	AC	129554948
rs10451555	COL4A3	AG	228167560
rs10452633	LAMA4	CT	112442767
rs10458655	P4HA1	AG	74809596
rs10461161	SPARCL1	AC	88404126
rs10463021	ADAM19	CT	156869405
rs10464068	COL23A1	AG	177711520
rs10464069	COL23A1	AG	177790952
rs10469935	EFEMP1	AC	56154316
rs10470990	NPNT	CT	106821578

rs10476093	ADAM19	AC	157007001
rs10479623	COL23A1	GT	177768048
rs10482979	ADAMTS5	CT	28316282
rs10487008	ADAM22	CT	87781111
rs10487009	ADAM22	AG	87783166
rs10487254	COL1A2	AC	94046199
rs10489328	TNN	GT	175113118
rs10489358	DPT	CT	168654873
rs10489359	DPT	AG	168658544
rs10489360	DPT	AG	168667989
rs10491806	ECM2	AG	95284212
rs10492497	COL4A1	CT	110831866
rs10492498	COL4A1	CT	110850523
rs10493856	TGFBR3	CT	92334749
rs10493859	TGFBR3	AC	92273644
rs10493860	TGFBR3	CT	92212703
rs10493986	COL11A1	CT	103570127
rs10493988	COL11A1	AG	103566156
rs10494553	LAMC1	AG	183115952
rs10494584	PRG4	CT	186290521
rs10494841	FMOD	CT	203328740
rs10495562	ADAM17	CT	9631340
rs10497699	COL5A2	CT	190032578
rs10498214	COL4A3	AG	228064180
rs10498215	COL4A3	CT	228062729
rs10498441	NID2	AG	52544224
rs10499146	LAMA2	AG	129330021
rs10499156	LAMA2	AG	129688123
rs10499159	LAMA2	AC	129781192
rs10500789	SPON1	GT	14031713
rs10510150	ADAM12	AC	127788934
rs10511316	CCDC80	AG	112347079
rs10514259	VCAN	AG	82879340
rs10514815	ECM2	AC	95256695
rs10517873	TLL1	AG	166850443
rs10519177	FBN1	AG	48757195
rs10519692	LOX	CT	121395749
rs10519694	LOX	CT	121407219
rs10520136	THBS1	AG	39864909
rs10521323	MMP2	AG	55481221
rs10521518	COL4A5	AG	107734573
rs10736104	TLL2	CT	98122334
rs10741636	SPON1	CT	14169984
rs10745384	COL5A1	CT	137662523
rs10748672	TLL2	CT	98117061
rs10751548	ADAM12	AG	128053597
rs10752893	LAMC1	CT	183046261
rs10752897	LAMC1	CT	183071024

rs10753939	FMOD	CT	203318731
rs10754833	NID1	CT	236184931
rs10763902	ITGB1	CT	33218076
rs10766169	SPON1	CT	14225465
rs10766177	SPON1	CT	14255818
rs10776897	COL5A1	AG	137578978
rs10783003	TGFBR3	CT	92195746
rs10791595	MMP10	CT	102635501
rs10794073	ADAM12	AC	127945261
rs10794075	ADAM12	CT	127951826
rs10794077	ADAM12	AG	127971058
rs10794080	ADAM12	CT	128053754
rs10794081	ADAM12	AC	128079595
rs10794082	ADAM12	CT	128085765
rs10798331	TNN	AG	175034437
rs10800913	FMOD	AG	203315893
rs10803234	NID1	AG	236189961
rs10808506	COL14A1	AG	121066470
rs10808507	COL14A1	AG	121166429
rs10817700	TNC	AG	117775803
rs10819542	COL15A1	AG	101738188
rs10827164	ITGB1	CT	33232628
rs10827167	ITGB1	CT	33277782
rs10832160	SPON1	AG	14039840
rs10832164	SPON1	CT	14048480
rs10832223	SPON1	CT	14185693
rs10832235	SPON1	AG	14244240
rs10851243	COL4A2	AC	111067499
rs10856792	MATN3	AG	20208412
rs10858265	COL5A1	CT	137543874
rs10859110	LUM	AG	91504845
rs10865714	FBLN2	AG	13600524
rs10874976	TGFBR3	AG	92267272
rs10874980	TGFBR3	AG	92283899
rs10882807	TLL2	CT	98268119
rs10901530	ADAM12	AC	127760446
rs10901534	ADAM12	AG	127830613
rs10901549	ADAM12	CT	127862926
rs10901552	ADAM12	CT	127868383
rs10901559	ADAM12	CT	127898686
rs10901580	ADAM12	CT	127962222
rs10901587	ADAM12	CT	128001759
rs10901589	ADAM12	CT	128005524
rs10901590	ADAM12	CT	128008557
rs10901596	ADAM12	AG	128021575
rs10901597	ADAM12	CT	128025384
rs10901601	ADAM12	CT	128045741
rs10901606	ADAM12	CT	128075882

rs10903227	COL23A1	CT	177988888
rs10908826	ADAMTS4	CT	161172525
rs10911203	LAMC1	AG	183004076
rs10911215	LAMC1	CT	183029613
rs10911233	LAMC1	AG	183052585
rs10912899	TNN	AG	175120715
rs10918959	DPT	GT	168667162
rs10920579	CHI3L1	AG	203158972
rs10920616	FMOD	AG	203318227
rs10929225	COL6A3	AG	238227776
rs10933170	COL4A3	GT	228078194
rs10933172	COL4A3	GT	228121147
rs10933175	COL4A3	AG	228133512
rs10945405	THBS2	CT	169621136
rs10949834	ELN	AG	73480569
rs10952902	ADAM22	AG	87573611
rs10952914	ADAM22	CT	87759483
rs10953329	PLOD3	CT	100865847
rs10955146	MATN2	AG	99017548
rs10982516	TNC	AG	117845611
rs10982521	TNC	CT	117853103
rs10982536	TNC	GT	117869417
rs10982549	TNC	AC	117889530
rs10988495	COL15A1	AG	101755900
rs11009157	ITGB1	AG	33242401
rs11023034	SPON1	AG	13976643
rs11023045	SPON1	CT	14015660
rs11023052	SPON1	CT	14042019
rs11023151	SPON1	AG	14246099
rs11069830	COL4A1	AC	110821132
rs11071829	CILP	CT	65493667
rs11071830	CILP	GT	65510624
rs11073813	ACAN	AG	89357446
rs11077405	LGALS3BP	CT	76969882
rs11080994	EMILIN2	CT	2897993
rs11080995	EMILIN2	CT	2900028
rs11100626	TLL1	AG	166827464
rs11103451	COL5A1	CT	137529221
rs11103479	COL5A1	CT	137610423
rs11103509	COL5A1	CT	137654103
rs11103534	COL5A1	GT	137700749
rs11103544	COL5A1	CT	137735043
rs11103545	COL5A1	GT	137739980
rs11105995	LUM	GT	91503261
rs11106030	DCN	AC	91575154
rs11130199	DAG1	CT	49538799
rs11134766	ADAM19	CT	156908317
rs11150610	ITGAM	AC	31334236

rs11153342	LAMA4	CT	112445106
rs11153346	LAMA4	CT	112534163
rs11153350	LAMA4	AG	112578384
rs11154461	LAMA2	GT	129368803
rs11154474	LAMA2	AG	129732674
rs11157873	NID2	AC	52525611
rs11157874	NID2	CT	52541543
rs11164624	COL11A1	CT	103336601
rs11164647	COL11A1	CT	103432687
rs11164658	COL11A1	GT	103499896
rs11164663	COL11A1	AC	103548497
rs11164665	COL11A1	AG	103551918
rs11164670	COL11A1	CT	103564540
rs11165262	TGFBR3	CT	92153974
rs11165354	TGFBR3	AC	92194322
rs11165489	TGFBR3	AG	92248274
rs11165594	TGFBR3	GT	92302369
rs11168337	COL2A1	AC	48376530
rs11168338	COL2A1	AC	48376969
rs11168353	COL2A1	CGT	48408212
rs11188736	TLL2	AG	98118257
rs11188741	TLL2	AG	98148857
rs11188748	TLL2	GT	98166356
rs11188749	TLL2	AG	98173991
rs11188751	TLL2	AG	98175291
rs11188758	TLL2	AG	98206115
rs11188775	TLL2	AG	98226956
rs11188795	TLL2	AG	98254309
rs11188796	TLL2	CT	98254356
rs11188799	TLL2	CT	98260206
rs11236458	SERPINH1	CT	75278666
rs11244772	ADAM12	AG	127711296
rs11244776	ADAM12	AC	127719180
rs11244777	ADAM12	AG	127719213
rs11244795	ADAM12	AG	127754286
rs11244796	ADAM12	CT	127756280
rs11244813	ADAM12	AG	127794080
rs11244841	ADAM12	CT	127834566
rs11244843	ADAM12	AG	127836991
rs11244849	ADAM12	GT	127845432
rs11244857	ADAM12	AG	127851943
rs11244887	ADAM12	GT	127902527
rs11244909	ADAM12	CT	127944593
rs11244911	ADAM12	AG	127946858
rs11244923	ADAM12	CT	127953451
rs11244924	ADAM12	AC	127955793
rs11244948	ADAM12	CT	127998826
rs11244955	ADAM12	AG	128005914

rs11244965	ADAM12	AG	128037247
rs11244969	ADAM12	AC	128068527
rs11244973	ADAM12	CT	128070801
rs11244975	ADAM12	AG	128075119
rs11247975	SPON2	GT	1165130
rs11249547	COL23A1	GT	177918571
rs11264300	ADAM15	AC	155019710
rs11264305	ADAM15	AG	155033572
rs11264306	ADAM15	AC	155043325
rs11465283	ADAM19	CT	156958127
rs11466561	TGFBR3	CT	92314842
rs11466782	ADAM19	AG	156921955
rs11547635	TIMP3	CT	33253292
rs11559201	MATN2	CT	99006748
rs11576557	TGFBR3	CT	92323809
rs11590043	DPT	AC	168692615
rs11590845	NID1	AC	236170665
rs11591508	ITGB1	CT	33284653
rs11597420	TLL2	AG	98151328
rs11603589	SPON1	AG	14217874
rs11606345	SPON1	CT	14218697
rs11626701	SERPINA12	AG	94954368
rs11630178	ACAN	CT	89373839
rs11632435	ACAN	AG	89347327
rs11633371	ACAN	GT	89356832
rs11635140	FBN1	CT	48782563
rs11637353	CSPG4	GT	76014429
rs11640811	MMP2	CT	55442880
rs11643163	MMP2	AG	55498659
rs11643666	MMP2	AG	55490089
rs11646699	MMP2	AG	55464722
rs11674048	COL6A3	CT	238223858
rs11677456	COL6A3	CT	238265531
rs11681705	MATN3	AG	20202811
rs11690358	COL6A3	CT	238244781
rs11694554	COL4A3	CT	228085517
rs11695502	EFEMP1	CT	56094999
rs11702367	ITGB2	AG	46335761
rs11702425	COL18A1	CT	46908355
rs11702782	COL18A1	AG	46848589
rs11704261	TIMP3	GT	33206863
rs11712980	FBLN2	AG	13600056
rs11730681	NPNT	AG	106922960
rs11738131	COL23A1	AC	177655194
rs11740086	COL23A1	CT	177755543
rs11742401	ADAM19	CT	156953340
rs11744096	COL23A1	CT	177707274
rs11745387	SPARC	AG	151059231

rs11745722	COL23A1	AG	177746863
rs11746584	COL23A1	CT	177667642
rs11746606	ADAM19	CT	156962499
rs11746864	COL23A1	AG	177838868
rs11747938	SPARC	CT	151037329
rs11749965	ADAM19	CT	156997414
rs11750135	ADAM19	CT	156957113
rs11750519	ADAM19	CT	156872416
rs11756439	THBS2	ACGT	169648030
rs11758161	THBS2	CT	169651344
rs11764718	COL1A2	AG	94059317
rs11766345	LAMB1	GT	107611650
rs11768465	PCOLCE	CT	100198386
rs11775186	BMP1	GT	22058122
rs11777932	BMP1	AC	22033435
rs11779647	MATN2	AC	98987643
rs11782639	COL14A1	AG	121369431
rs11784449	MATN2	GT	99015018
rs11785807	MATN2	CT	99011698
rs11787664	TNC	CT	117781656
rs11789200	TNC	AG	117812791
rs11802668	PLOD1	CT	12000670
rs11804204	DPT	AG	168695047
rs11809524	COL11A1	CT	103459537
rs11813508	ADAM12	CT	127742994
rs11817398	P4HA1	AG	74759871
rs11847418	NID2	AG	52490630
rs11853604	ACAN	GT	89388335
rs11856834	CILP	CT	65496287
rs11859163	MMP2	GT	55502376
rs11861251	ITGAM	CT	31289396
rs11871320	LGALS3BP	CT	76964531
rs11871490	MFAP4	CT	19296893
rs11884740	COL4A3	AG	228093667
rs11884770	COL4A3	CT	228086920
rs11891192	ITGAV	GT	187546583
rs11891922	ADAM17	AG	9649966
rs11896721	COL6A3	AG	238284107
rs11900265	ADAM17	CT	9680013
rs11902058	COL3A1	AC	189862808
rs11909037	ADAMTS1	CT	28226552
rs11923433	PLOD2	AG	145886725
rs11924163	TIMP4	AG	12210153
rs11925421	PLOD2	AG	145888162
rs11928651	ITGB5	CT	124577555
rs11933466	NPNT	AG	106812732
rs11951889	ADAM19	CT	156876784
rs11956401	COL23A1	AG	177848109

rs11958296	COL23A1	AG	177755179
rs11963652	LAMA2	AG	129551015
rs11966235	THBS2	CT	169626830
rs11988416	COL14A1	AG	121333937
rs11996075	MATN2	CT	98910287
rs11998946	COL5A1	CT	137679703
rs12001981	COL15A1	AG	101820545
rs12019361	ADAM22	AG	87573647
rs12023499	ADAM15	CT	155031376
rs12027195	NID1	GT	236187011
rs12046389	COL11A1	AC	103409100
rs12050562	FBN1	CT	48701228
rs12052967	COL6A3	AG	238299189
rs12073224	TGFBR3	CT	92200963
rs12076549	TGFBR3	AC	92201699
rs12077300	FMOD	CT	203317708
rs12082710	TGFBR3	CT	92155337
rs12083818	TNN	CT	175083952
rs12094153	TNN	AG	175094025
rs12098279	ADAM12	AG	127753985
rs12101282	FBLN5	AG	92342442
rs12114940	BMP1	GT	22048790
rs12118365	COL11A1	CT	103438795
rs12121498	COL11A1	AG	103380128
rs12123436	TGFBR3	AG	92215089
rs12123883	CHI3L1	CT	203164842
rs12126497	DPT	GT	168672858
rs12128607	PRG4	CT	186275564
rs12131957	COL11A1	CT	103467223
rs12133988	DPT	CT	168678324
rs12134934	PRG4	CT	186281400
rs12138977	COL11A1	CT	103393457
rs12141988	COL11A1	AG	103371544
rs12142096	COL11A1	CT	103362909
rs12145872	COL11A1	CT	103371652
rs12146041	TNN	AG	175102161
rs12147719	SERPINA12	AG	94979268
rs12153855	TNXB	CT	32074804
rs12165359	TIMP3	CT	33249271
rs12184772	COL4A1	AG	110847746
rs12190465	LAMA2	AG	129383680
rs12190908	LAMA4	CT	112527786
rs12192613	LAMA2	CT	129199783
rs12192658	LAMA4	AG	112504240
rs12193446	LAMA2	AG	129820038
rs12195039	LAMA4	CT	112580969
rs12195178	LAMA2	CT	129793012
rs12195187	LAMA2	AG	129519893

rs12198087	LAMA4	AG	112534860
rs12198173	TNXB	AG	32026808
rs12202008	LAMA2	CT	129375673
rs12203042	LAMA2	CT	129817953
rs12204892	LAMA4	CT	112431283
rs12205363	LAMA2	CT	129834629
rs12206487	LAMA2	CT	129619410
rs12208401	LAMA2	CT	129620506
rs12213754	LAMA2	AG	129439995
rs12215389	COL12A1	AG	75889208
rs12215657	LAMA2	CT	129684068
rs12215704	LAMA2	CT	129519697
rs12216916	COL15A1	CT	101813506
rs12228854	COL2A1	GT	48396920
rs12235159	TNC	CT	117841938
rs12243508	ADAM12	AG	127997728
rs12251014	ADAM12	CT	127958529
rs12264492	ADAM12	AG	127854246
rs12271585	SPON1	GT	14006425
rs12272341	MMP10	AG	102644601
rs12277421	SERPINH1	AG	75267648
rs12283632	SPON1	AG	14058278
rs12290253	MMP10	CT	102642261
rs12300271	COL2A1	CT	48374616
rs12332199	VCAN	CT	82786194
rs12333245	TNXB	AG	32019769
rs12352939	COL15A1	CT	101786106
rs12354990	ADAM12	GT	128037499
rs12365614	SPON1	AC	14093889
rs12404952	TGFBR3	CT	92215725
rs12409606	NID1	CT	236220574
rs12411075	NID1	CT	236182924
rs12413925	TLL2	CT	98208861
rs12415694	ADAM12	CT	128045927
rs12416625	ADAM12	CT	127890797
rs12423250	COL2A1	CT	48389245
rs12433651	SERPINA12	AG	94964789
rs12434141	SERPINA12	AG	94947318
rs12437465	LOXL1	CT	74243246
rs12439075	ACAN	CT	89355911
rs12440667	LOXL1	CT	74231439
rs12442211	LOXL1	AG	74245675
rs12454179	EMILIN2	AG	2875255
rs12463454	PDIA6	AG	10958235
rs12468719	COL4A3	AG	228140460
rs12470793	COL4A3	CT	228046777
rs12473402	ADAM17	AC	9632718
rs12476069	COL6A3	CT	238321317

rs12477499	COL3A1	AG	189833034
rs12478941	MATN3	GT	20218658
rs12483377	COL18A1	AG	46931109
rs12483553	COL18A1	AG	46926093
rs12487905	ITGB5	CT	124521035
rs12488326	ITGB5	AG	124490088
rs12513380	TLL1	AG	166830364
rs12519834	COL23A1	AG	177802373
rs12523864	LAMA2	CT	129827116
rs12526196	CTGF	CT	132263476
rs12532878	PCOLCE	AG	100212254
rs12541735	COL14A1	CT	121183650
rs12541737	MATN2	AC	98972528
rs12545308	COL14A1	CT	121385167
rs12551466	COL5A1	AG	137737438
rs12554098	COL5A1	AG	137573609
rs12563833	TNN	AG	175109517
rs12565776	LAMC1	AG	183116531
rs12569424	TLL2	CT	98258871
rs12571387	ADAM12	AG	127769862
rs12573768	P4HA1	AG	74764074
rs12574121	SPON1	CT	14254796
rs12586793	FBLN5	AG	92401634
rs12589592	FBLN5	AG	92377802
rs12620429	ITGAV	AC	187489647
rs12620653	COL4A3	CT	228086059
rs12622093	COL6A3	AG	238283605
rs12622722	COL6A3	AG	238269120
rs12626746	COL6A2	CT	47548011
rs12642840	TLL1	GT	166955001
rs12643464	SPARCL1	CT	88438520
rs12652833	COL23A1	CT	177679728
rs12653308	VCAN	AG	82858828
rs12658197	VCAN	AG	82884488
rs12660510	LAMA2	AG	129666314
rs12662729	LAMA2	CT	129717442
rs12664291	LAMA2	CT	129471063
rs12665286	THBS2	AG	169609256
rs12665573	THBS2	AG	169625790
rs12668754	COL1A2	AG	94059236
rs12669586	ADAM22	GT	87666274
rs12676720	MATN2	CT	98927699
rs12678374	COL14A1	AC	121319783
rs12683163	TNC	CT	117815011
rs12684637	COL5A1	CT	137633543
rs12686426	COL5A1	CT	137642577
rs12721420	COL2A1	CT	48358794
rs12721427	COL2A1	CT	48368541

rs12722864	TGFBR3	CT	92191534
rs12733054	DPT	CT	168689602
rs12739316	LAMC1	AG	183052080
rs12751148	TGFBR3	CT	92190072
rs12757745	FMOD	AG	203308968
rs12759840	NID1	AG	236150576
rs12762349	ADAM12	GT	127825898
rs12767127	ADAM12	AG	127848780
rs12769591	P4HA1	AG	74808045
rs12777779	ADAM12	CT	127860067
rs12778749	ADAM12	CT	127846669
rs12795982	SPON1	AG	14114318
rs12799462	SPON1	AC	13977738
rs12801550	SPON1	CT	13993754
rs12868126	COL4A1	AG	110822524
rs12868443	COL4A2	CT	111066147
rs12870273	POSTN	CT	38173439
rs12871092	POSTN	AG	38159633
rs12873113	COL4A2	GT	111155230
rs12873154	COL4A1	AG	110920852
rs12874003	COL4A1	AG	110951315
rs12874003	COL4A2	AG	110951315
rs12894295	NID2	AG	52497718
rs12904518	LOXL1	AC	74247944
rs12905452	ACAN	AC	89371423
rs12906911	FBN1	AC	48828161
rs12915677	FBN1	CT	48864797
rs12918370	MMP2	AG	55424127
rs12924764	MMP2	AG	55498049
rs12931375	MMP2	GT	55467633
rs12945599	COL1A1	CT	48255183
rs12969432	EMILIN2	AG	2899428
rs12992087	COL6A3	CT	238324700
rs13005714	COL5A2	CT	189999011
rs13006483	ITGAV	GT	187522750
rs13010821	GPC1	CT	241416038
rs13015794	GPC1	CT	241369051
rs13021572	COL4A3	AG	228090768
rs13024858	COL5A2	GT	189981212
rs13028299	COL4A3	AG	228091661
rs13028444	COL3A1	AC	189876138
rs13030208	GPC1	GT	241368464
rs13048083	ADAMTS5	CT	28286853
rs13050660	COL6A2	CT	47546244
rs13050770	ITGB2	CT	46335401
rs13058539	FBLN1	CT	45962522
rs13079599	CCDC80	AG	112346521
rs13082230	ITGB5	AG	124478734

rs13110600	TLL1	GT	166810059
rs13125117	NPNT	CT	106909698
rs13136952	TLL1	CT	166833808
rs13153507	VCAN	AC	82845311
rs13159176	COL23A1	CT	177899270
rs13164785	VCAN	GT	82861400
rs13178731	COL23A1	AG	177811754
rs13182062	COL23A1	CT	177803040
rs13187034	COL23A1	CT	177979374
rs13187323	COL23A1	CT	177902122
rs13199524	TNXB	CT	32066765
rs13201278	COL12A1	AG	75915157
rs13204236	LAMA2	AG	129692686
rs13216682	LAMA2	CT	129394663
rs13245672	ADAM22	AG	87688046
rs13257404	COL14A1	AG	121125111
rs13257482	BMP1	AG	22059606
rs13276675	COL14A1	CT	121178772
rs13288677	COL5A1	AG	137581319
rs13294483	COL5A1	AG	137675994
rs13297317	COL5A1	AG	137701873
rs13302368	COL15A1	AG	101807566
rs13306275	COL3A1	AG	189855166
rs13311608	FKBP9	AG	32999601
rs13353878	ADAM19	AG	156960581
rs13383261	COL5A2	CT	189913748
rs13392414	COL4A3	AG	228060884
rs13419630	COL4A3	AG	228056218
rs13422838	ITGAV	CT	187502846
rs13425379	COL3A1	AG	189844599
rs13429247	PDIA6	AG	10960529
rs13429913	COL4A3	CT	228058913
rs13431876	COL6A3	AG	238316009
rs13432972	FN1	AC	216248460
rs13433666	FBLN1	CT	45902119
rs13438950	COL14A1	CT	121369401
rs16833075	NID1	GT	236157277
rs16833089	NID1	CT	236162449
rs16835998	ITGB5	CT	124500094
rs16836078	ITGB5	CT	124548599
rs16836080	ITGB5	AG	124548684
rs16859850	CCDC80	GT	112339845
rs16859897	CCDC80	AG	112362600
rs16893529	COL14A1	AG	121101436
rs16893536	COL14A1	CT	121103751
rs16893621	COL14A1	AG	121179736
rs16893954	COL14A1	AG	121371823
rs16896468	MATN2	AG	98974256

rs16896493	MATN2	AGT	98994920
rs16896564	MATN2	CT	99032925
rs16918081	COL15A1	CT	101697350
rs16918099	COL15A1	AG	101715598
rs16918124	COL15A1	CT	101761125
rs16918167	COL15A1	GT	101815339
rs16932062	TNC	CT	117776052
rs16932078	TNC	CT	117796986
rs16932160	TNC	AC	117829987
rs16943929	EMILIN2	AG	2871811
rs16943989	EMILIN2	AG	2892474
rs16944003	EMILIN2	CT	2899324
rs16955194	MMP2	AG	55505328
rs16960886	FBN1	AG	48699486
rs16961033	FBN1	AG	48795877
rs16961065	FBN1	CT	48806539
rs16961239	FBN1	AG	48906457
rs16969364	THBS1	AG	39885470
rs16975420	COL4A1	AC	110813991
rs16975617	COL4A1	CT	110867408
rs16994329	FBLN1	CT	45997852
rs17012774	SPARCL1	CT	88443470
rs17012782	SPARCL1	AG	88443685
rs17018718	LUM	CT	91498199
rs17018731	LUM	CT	91499367
rs17036413	NPNT	CT	106894867
rs17036476	NPNT	AG	106922246
rs17038334	FBLN2	AG	13647142
rs17047075	TLL1	AC	166839266
rs17047205	TLL1	AG	166953579
rs17047207	TLL1	AG	166956652
rs17047228	TLL1	CT	166997752
rs17047248	TLL1	AG	167011894
rs17054657	ADAM19	CT	156927875
rs17054692	ADAM19	AG	156962401
rs17057149	LAMA2	CT	129644548
rs17057158	LAMA2	CT	129670548
rs17073563	LAMA4	CT	112527772
rs17081208	COL23A1	AG	177849300
rs17081220	COL23A1	CT	177852582
rs17090997	SERPINA12	AG	94986912
rs17094919	SERPINA12	CT	94984522
rs17111762	TLL2	CT	98141748
rs17111841	TLL2	AG	98179762
rs17122926	MMP14	GT	23299915
rs17122962	MMP14	AG	23302565
rs17124884	NID2	AG	52482698
rs17124893	NID2	AG	52485197

rs17124972	NID2	AG	52521893
rs17125001	NID2	CT	52541199
rs17127492	COL11A1	AG	103576364
rs17127649	FBLN5	CT	92339519
rs17131536	TGFBR3	CT	92181478
rs17131552	TGFBR3	AG	92224815
rs17131553	TGFBR3	AG	92226887
rs17134462	PPIA	AG	44845032
rs17145990	ELN	CT	73438515
rs17150369	ADAM22	CT	87839690
rs17154105	ADAM12	AC	127733872
rs17154306	ADAM12	AG	127829304
rs17154587	ADAM12	CT	127946649
rs17154643	ADAM12	AG	127964859
rs17154787	ADAM12	AG	128083065
rs17155554	ADAM12	AC	127936662
rs17178474	FBLN2	CT	13584980
rs17201864	ACAN	CT	89420974
rs17205951	VCAN	GT	82848324
rs17232065	MMP2	CT	55425383
rs17237080	COL14A1	CT	121295744
rs17241017	MMP2	AG	55485757
rs17252755	ADAM22	CT	87573612
rs17282085	ITGB5	AG	124521263
rs17292943	COL3A1	AG	189847300
rs17293152	MMP10	AG	102633134
rs17293642	MMP1	CT	102654928
rs17293642	MMP10	CT	102654928
rs17296289	ITGB1	AG	33260699
rs17299191	MMP2	CT	55472083
rs17300655	MMP2	AC	55491049
rs17301608	MMP2	CT	55518610
rs17319250	PLOD3	CT	100861213
rs17342005	ADAM22	CT	87800082
rs17352842	FBN1	CT	48694211
rs17357592	COL6A2	CT	47542779
rs17358566	COL3A1	CT	189837995
rs17359286	MMP10	GT	102643718
rs17364665	FBN1	AC	48884829
rs17365477	PDIA6	CT	10970124
rs17367956	COL4A3	AG	228064828
rs17388474	TLL2	AG	98129058
rs17455729	PDIA6	AG	10928250
rs17463112	SPON1	CT	14001955
rs17505781	TLL1	CT	166967504
rs17513917	TGFBR3	CT	92205197
rs17516906	FN1	AG	216279273
rs17517598	COL4A1	GT	110825264

rs17517971	COL4A1	AG	110828119
rs17556665	SPON1	GT	14098482
rs17564689	FBLN1	AG	46006110
rs17571088	TGFBR3	AG	92146890
rs17574203	TGFBR3	AC	92265144
rs17576372	TGFBR3	CT	92366174
rs17588591	COL4A1	GT	110808738
rs17589948	COL23A1	AG	177904577
rs17590344	TLL1	AG	166960363
rs17590882	ADAM17	AG	9661775
rs17599222	ADAM19	GT	156817266
rs17633210	THBS1	AC	39900566
rs17641413	COL23A1	CT	177773700
rs17648414	COL23A1	CT	177851690
rs17659250	ADAM19	CT	156940873
rs17684676	ADAM12	AG	127951798
rs17684713	ADAM12	AC	127952874
rs17689892	TLL1	AC	166927050
rs17689964	TLL1	AG	166946775
rs17711860	COL15A1	AG	101800106
rs17718324	SPARC	AG	151058179
rs17742838	LAMA2	CT	129681346
rs17752721	LAMA2	CT	129820505
rs17752900	SERPINA12	CT	94983794
rs17752932	SERPINA12	AG	94986871
rs17753252	LAMA2	AG	129841984
rs17759651	ADAM12	CT	128066982
rs17783485	LAMA2	AG	129427787
rs17783615	COL12A1	CT	75878185
rs17799083	LAMA2	AC	129673136
rs17802266	SPON2	AC	1158991
rs17805293	FBLN5	CT	92369592
rs17805775	CILP	CT	65505038
rs17819305	TNC	CT	117816230
rs17825644	SERPINA12	CT	94944112
rs17829645	MATN2	CT	98954739
rs17833457	COL14A1	GT	121276075
rs35013643	ITGB2	AG	46320283
